# Global, Regional, and National Burden of Child Growth Failure, 1990–2021: A Systematic Analysis for the Global Burden of Disease Study 2021

**DOI:** 10.3390/nu17071185

**Published:** 2025-03-28

**Authors:** Kelly Lin, Nicholas Buys, Jun Zhou, Yanfei Qi, Jing Sun

**Affiliations:** 1 Rural Health Research Institute, Charles Sturt University, Bathurst, NSW 2800, Australia; kelly.lin@griffithuni.edu.au; 2 School of Medicine and Dentistry, Griffith University, Gold Coast, QLD 4215, Australia; 3 School of Health Science and Social Work, Griffith University, Brisbane, QLD 4215, Australia; n.buys@griffith.edu.au; 4 School of Information and Technology, Griffith University, Nathan, QLD 4215, Australia; jun.zhou@griffith.edu.au; 5 Centenary Institute, The University of Sydney, Sydney, NSW 2050, Australia; 6 Data Science Institute, University of Technology Sydney, Sydney, NSW 2000, Australia

**Keywords:** growth failure, stunting, wasting, chronic malnutrition

## Abstract

**Background/Objectives**: Child growth failure is a manifestation of chronic malnutrition expressed in stunting, wasting, and underweight in children. This study aimed to analyze global trends in child growth failure disease burden and mortality across children of all age groups on a global, regional, and national level. **Methods**: This cross-sectional study utilized data from the 1990 and 2021 Global Burden of Disease (GBD) study. Growth failure Disability-adjusted life years (DALYs), years lived with a disability (YLDs), and mortality in children under 20 years of age were analyzed. Average annual percentage change (AAPC) was calculated to determine and identify improvements in growth failure disease burden and mortality in the past 30 years. **Results**: Greatest reduction in growth failure DALYs (AAPC = −0.96, 95% CI = −0.97 to −0.95), YLDs (AAPC = −0.73, 95% CI = −0.77 to −0.66) and mortality rate (AAPC = −0.96, 95% CI = −0.97 to −0.95) in children under 5 years of age was observed in high-middle SDI countries. In contrast, improvements in the number of growth failure DALYs (AAPC = −0.64, 95% CI = −0.76 to −0.53), YLDs (AAPC = −0.21, 95% CI = −0.25 to −0.13) and mortalities (−0.57, 95% CI = −0.59 to −0.52) are less pronounced in regions with low SDI scores. Improvements in disease burden and mortality are reduced in older age groups, with the lowest reduction observed in children 15–19 years old. **Conclusions**: Barriers hindering the delivery of nutritional supplements and access to quality healthcare in regions with low SDI scores need to be overcome to address the disproportionately high numbers of growth failure DALYs, YLDs, and mortalities in regions with low SDI.

## 1. Introduction

Malnutrition is a critical risk factor associated with developmental delays and premature mortality [1]. Different forms of malnutrition exist including specific micronutrient deficiencies, excess or imbalance in nutrient intake, and undernutrition [2]. This study specifically focuses on growth failure associated with chronic undernutrition in children, preventing adequate child development and growth. Child growth failure is a manifestation of chronic undernutrition expressed in stunting, wasting, and underweight in children. Stunting results from chronic undernutrition causing linear growth retardation, while wasting refers to inadequate nutrition over a shorter period, resulting in weight that is too thin for height, due to rapid weight loss or failure to gain weight [3]. Globally, malnutrition has contributed to around 45% of deaths for children under 5 years of age [4]. Chronic malnutrition has long-term effects with greater risks of cognitive and physical development impairments that can lead to worse educational attainment and reduced income in adulthood. In line with the detrimental effects malnutrition has on child well-being and development, ending all forms of malnutrition, including growth failure (stunting and wasting) by 2025 has been identified as the second target in the United Nation’s Sustainable Development Goals (SDGs). Furthermore, to ensure the second SDG can be met, the World Health Organisation (WHO) has endorsed a comprehensive plan to improve maternal, and childhood nutrition with aims to achieve six global nutritional targets by 2025. Two of these six goals aim to address growth failure, including achieving a 40% reduction in stunting in children under 5 and reducing and maintaining childhood wasting to less than 5% by 2025 [5]. However, past reviews have revealed significant differences in the progress towards achieving nutritional targets between countries of different sociodemographic indices (SDI) [6].

Global and national reports have revealed significant heterogeneity in the prevalence of growth failure between countries and age groups. Malnutrition and its manifestations including growth failure, increase the risk of premature mortality due to worse immunity, with increased risk of acquiring infectious diarrheal and respiratory diseases with adverse outcomes [7]. Children in low to middle-income countries (LMICs) with a low socio-demographic index (SDI) are particularly vulnerable to malnutrition and growth failure due to socioeconomic disadvantages, including food insecurity, poor nutrition, worse quality of care, and inadequate access to healthcare services [6,8]. Worse maternal demographic factors such as limited education and poor maternal health also negatively influence feeding practices, rates of breastfeeding, use of nutritional supplements, and access to healthcare. Inequalities in available infrastructure, government initiatives, and individual demographic factors puts children in LMICs at higher risks of malnutrition and growth failure. Since child malnutrition is associated with access, and quality of healthcare and household demographic factors, child growth failures including stunting and wasting have been identified as a marker for child health inequalities [9]. Following the identification of high growth failure prevalence in children in LMICs, national policies and global financial aid have been dedicated to reducing healthcare and nutritional inequalities in LMICs with the aim of achieving SDG 2 of ending all forms of malnutrition [10]. To determine the progress towards achieving SDG, two trends in the prevalence of regional growth failure must be monitored and predicted. Thus, this study aims to compare changes in the regional prevalence of growth failure in children and adolescents from 1990 to 2019.

Most studies focus on children under 5 years of age, revealing long-term physical and cognitive impairments [6,11]. However, growth failure also affects adolescents. Adolescence is a critical period of rapid development with increased nutritional demands, making adolescents more susceptible to growth failure when nutritional needs are not met [12]. Growth failure during adolescence can be detrimental, as adolescent growth stunting and wasting have been associated with poor cognitive development, greater risk for obstetric complications in females, and diminished physical work capacity [13]. Despite the importance of nutrition during adolescence, a gap in adolescent nutritional data is present [13]. To the author’s knowledge, no previous study has identified or compared the trends of adolescent growth failure on a global level. Thus, this study aims to examine global and regional trends in growth failure in both children and adolescents under 20 years of age. Differences in the prevalence of growth failure of children and adolescents in different age groups will also be determined to identify the group at greatest risk.

## 2. Materials and Methods

### 2.1. Overview

This study follows the Guidelines for Accurate and Transparent Health Estimates Reporting (GATHER) statement and GBD protocol (Supplementary Materials). Further descriptions of the methodology for GBD estimation can be found elsewhere [14].

### 2.2. Case Definition and Input Data

The standard definitions provided by the GBD categorize the causes of growth failure into the following categories: deficiencies in height-for-age (stunting), weight-for-height (wasting), and weight-for-age (underweight) measured as standard deviations from the median of age- and sex-specific growth standards published by the WHO [5,10].

This cross-sectional study utilized data from the 2021 GBD study that modeled nonfatal disease burden using DisMod-MR version 2.1. This meta-analysis tool uses a compartmental model structure with different equations that help synthesize heterogeneous epidemiologic data for non-fatal disease, including growth failure analyzed in the current study. In line with WHO guidelines, growth failure was defined as children that are stunted, wasted, or underweight if their height-for-age, weight-for-height, or weight-for-age Z-score is more than 2 standard deviations below the standards established for a healthy population [13]. Further details, including International Classification of Diseases (ICD) codes for each disease, are provided in the Supplementary Materials.

This study utilized data on disease burden as measured by Disability-adjusted life years (DALYs) and Years Lived With Disability (YLDs), and mortality using age-standardized mortality rates (ASMRs). Only data from children under 20 years of age from 1990 to 2021 were extracted from the Global Health Data Exchange query tool. Data from 204 countries and territories were accessed. Data from the included countries were classified into 5 regions based on sociodemographic index (SDI) and 21 GBD regions according to geographical contiguity.

### 2.3. SDI

The sociodemographic index (SDI) is a measure that encompasses the economy as measured by lag distributed income (LDI) per capita, mean education for those 15 and older, and total under 25 fertility rates of nations, which can be used to represent a country’s social and economic development [15]. This index is used in Global Burden of Disease studies as outcomes measured by SDI correlate strongly with health outcomes. Based on the country’s SDI score, each country has been classified into one of the five categories—high, high-middle, middle, low-middle, and low. Growth failure DALYs, YLDs, and mortality were compared across all 5 SDI categories across all age groups.

### 2.4. Age Groups

Data from children under 20 years of age were extracted in this study. Children were further divided into 5 age groups, including under 5, 5 to 9 years, 10 to 14 years, 15 to 19 years, and a total under 20. Children under 5 years of age were specifically analyzed as younger children are at greater risk of malnutrition and its consequences. Children were then grouped in 5-year intervals spanning early to middle childhood and adolescence. Age-standardized results were also reported for the rate of DALYs, death, and YLDs.

### 2.5. Data Source

Data from the 2021 GBD study that modeled nonfatal disease burden using Dismod-MR version 2.1 was used for analysis in this study. We analyzed growth failure mortalities and growth failure disease burden through Disability-adjusted life years (DALYs) and Years Lived With Disability (YLDs). Data from children under 20 years of age from 1990 to 2021 from 204 countries and territories were included for analysis. Countries were further classified into five groups based on sociodemographic index (SDI) and 21 GBD regions according to geographical contiguity. Growth failure was the main outcome analyzed, analysis of other malnutrition outcomes including vitamin A deficiency, suboptimal breastfeeding, low birth weight, and short gestation have been described in other papers as part of the study series [16].

### 2.6. Statistical Analysis

This study followed GDP study protocols.

The statistical analysis methodology used in this paper can be found in our previously published papers [16].

### 2.7. Role of Funding Source

The study funder had no role in the study design, data collection, data analysis, data interpretation, report writing, or decision to submit the manuscript for publication.

## 3. Results

### 3.1. Global

**Figure 1:**
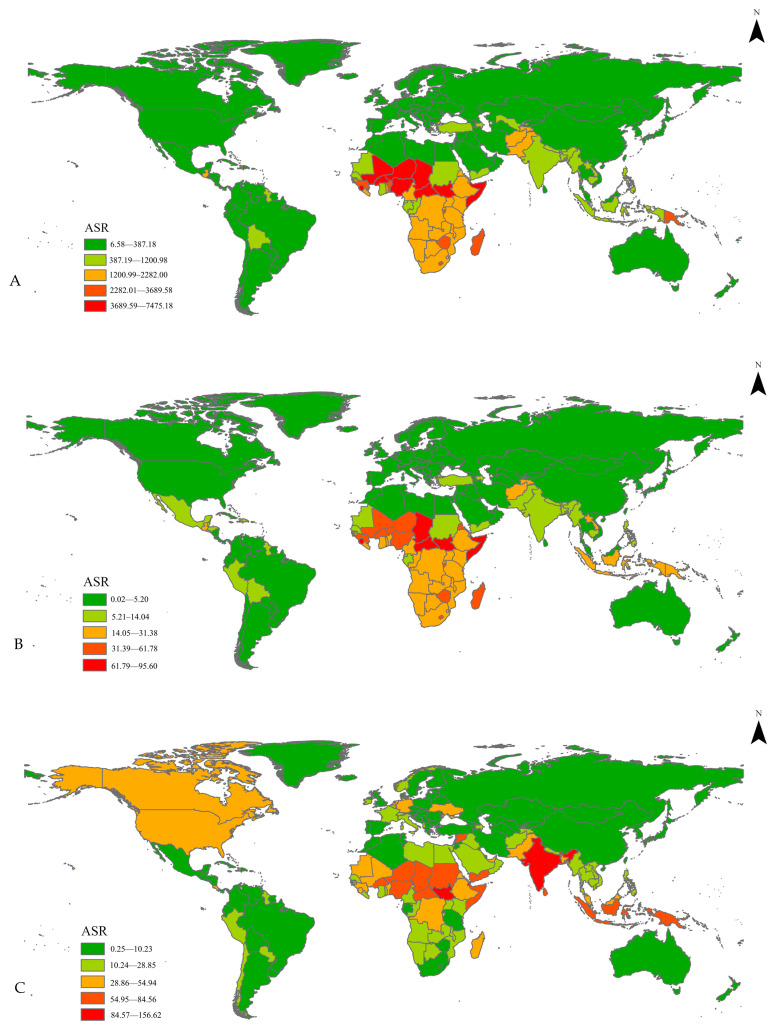
Age-standardized rate (ASR) of global growth failure (**A**) DALYs, (**B**) mortalities, (**C**) YLDs. ASR = age-standardized rate.

Table 1 and Figure 1 show the changes in global growth failure DALYs, YLDs, and mortality in children under 20 years of age from 1990 to 2021. Changes in growth failure disease burden and mortality in children across 5 SDI groups are also presented in Table 1.

Significant global reduction in DALYs, deaths, and YLDs with growth failure as a cause can be observed across all age groups and age-standardized results, indicating significant improvement in reduced disease burden and mortality due to growth failure. The most significant reduction in DALYs (AAPC number = −0.78, 95% CI = −0.85 to −0.71), deaths (AAPC number = −0.78, 95% CI = −0.85 to −0.72) and YLDs (AAPC number = −0.5, 95% CI = −0.52 to −0.44) are all observed in children under 5 years of age. The extent of improvement in growth failure DALYs, YLDs, and mortality are less pronounced in older age groups, with the least improvement in children in the 15–19 years age group.

### 3.2. Sociodemographic Index

Across 5 SDI groups, significant reductions in growth failure DALYs, YLDs, and mortality were observed across children in countries of all SDI levels. The greatest reduction in DALYs, YLDs (AAPC = −0.73, 95% CI = −0.77 to −0.66), and mortality rate (AAPC = −0.96, 95% CI = −0.97 to −0.95) in children under 5 years of age due to growth failure is observed in countries of the high-middle SDI group. From 1990 to 2021, in high-middle SDI countries, the number of DALYs reduced from 12,107,630.38 to 381,755.92 with an AAPC = −0.96 (95% CI = −0.97 to −0.95), the number of growth failure mortalities reduced from 136,510 to 3921.63 with an AAPC of −0.96 (95% CI = −0.97 to −0.95), and the number of YLDs reduced from 126,049.91 to 32,440.07 with an AAPC of −0.73 (95% CI = −0.77 to −0.61).

In contrast, the number of growth failure DALYs, YLDs, and mortalities are disproportionately higher in regions with low SDI scores, with less pronounced improvements in the past 30 years as well. From 1990 to 2021, the number of growth failure DALYs reduced from 125,311,062.81 to 45,382,461.21 with an AAPC of −0.64 (95% CI = −0.76 to −0.53), while the number of associated mortalities decreased from 1,433,045.77 to 505,070 with an AAPC of −0.64 (95% CI = −0.76 to −0.53) and the number of YLDs reduced from 491,077.48 to 729,825.13 with an AAPC of −0.21 (95% CI = −0.25 to −0.13). This suggested that the improvements in children’s growth failure in countries with low SDI scores are smaller. The rate of under-20 growth failure mortality was 87.27 per 100,000 children in low SDI regions, which is 33.3 higher than the growth failure mortality identified in high SDI regions of 2.62 per 100,000 people. Furthermore, like global trends, improvements in disease burden and mortality were reduced in older age groups, with the lowest reduction observed in children 15–19 years old across high-middle, middle, low-middle, and low SDI groups.

### 3.3. Regional

**Figure 2:**
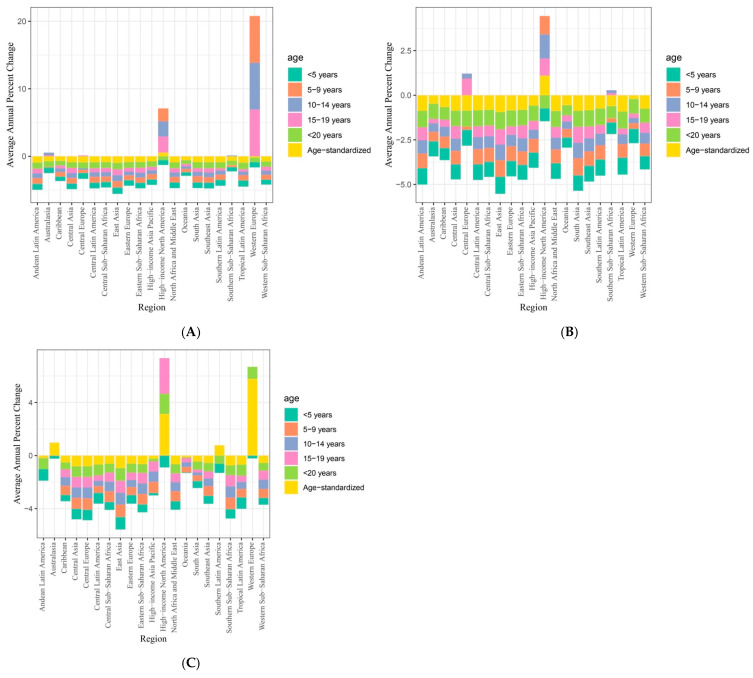
Regional 1990 to 2021 growth failure AAPC (**A**) DALYs, (**B**) mortalities, and (**C**) YLDs.

Countries were further divided into 21 regions based on their geographical location with age-standardized results displayed in Figure 2. Regional results on the disease burden and mortality of growth failure in under 20-year-old children are presented in total number and rate per 100,000 children in Table 2. Significant heterogeneity in improvements was found across countries from different regions. When assessed against the 2030 nutritional target of a 40% reduction in stunting in children under 5 years of age, countries in Oceania, Western Sub-Saharan Africa, Central Sub-Saharan Africa, and Eastern Sub-Saharan Africa region were at risk of not achieving this target.

In terms of mortality, the number of under 5 growth failure mortality decreased from 4602.19 to 4997.04 with an AAPC of −0.19 (95% CI = −0.4 to 0.05), indicating no significant change over time. In Western Sub-Saharan countries, the number of growth failures under 5 mortalities decreased from 6,660,961.77 to 338,547.65, with an AAPC of −0.47 (95% CI = −0.67 to −0.31). In contrast, East Asia showed the most significant improvement, with a decrease in the number of growth failures under 5 mortalities from 356,998.01 to 5286.23 (AAPC = −0.98, 95% CI = −0.99 to −0.97).

As shown in Table 3, in terms of disease burden, some regions showed an increase in under-5 growth failure while some regions showed minimal improvements. Disease burden measured by YLDs showed an increase in the number of failures in under 5 YLDs from 3435.8 to 8694.27 and a rate per 100,000 children from 349.46 to 449.44 in the Oceania region. Although the AAPC for number (0.82, 95% CI = −0.11 to 3.73) and rate (−0.06, 95% CI = −0.54 to 1.46) of growth failure YLDs for under 5 children in Oceania did not reach a level of statistical significance, absence of change indicates no improvement, meaning that Oceania countries at risk of not achieving the 2030 nutritional target of a 40% reduction in stunting as part of growth failure. Western Sub-Saharan countries also showed an increase in the number of under 5 growth failure YLDs from 121,827.84 to 357,020.29 (AAPC = 0.06, 95% CI = −0.05 to 0.5) and rate per 100,000 children from 341.51 to 446.52 (AAPC = −0.53, 95% CI = −0.33 to −0.58). Number and rate of under 5 growth failure YLDs in Central Sub-Saharan region increased from 33,192.21 to 68,447.07 (AAPC = −0.21, 95% CI = −0.38 to −0.09) and 308.77 to 324.91 (AAPC = −0.61, 95% CI = −0.69 to −0.55) respectively. Lastly, in the Eastern Sub-Saharan African region, the number of under-5 growth failures also increased from 97,223.27 to 166,416.92, with a non-significant AAPC of −0.32 (95% CI = −0.38 to 0.32).

### 3.4. National

National growth failure results were age-standardized and included both children and adolescents under 20 years of age. Detailed national growth failure burden of disease results are included in Supplementary Table S1. Similar to regional results, while significant reductions in growth failure burden were identified, the burden of growth failure morbidity and mortalities remained greatest amongst low SDI countries. Southeast Asia and the Sub-Saharan African region had the highest age-standardized rate of growth failure-associated mortalities and morbidities in 2021. In Sub-Saharan African countries such as Burkina Faso, Ghana, and Sierra Leone, the age-standardized rate of growth failure mortalities in 2021 was 128.45 (95% CI = 41.99 to 208.82), 26.84 (95% CI = 9.34 to 49.22), and 128.75 (95% CI = 55.60 to 209.28), respectively. In Southeast Asian countries such as Bangladesh, Cambodia, and Myanmar, growth failure mortalities were 13.71 (95% CI = 9.23 to 18.70), 22.59 (95% CI = 16.09 to 30.29), and 23.58 (95% CI = 14.65 to 32.69), respectively. This is significantly higher than the ASR in high SDI countries such as Australia with a growth failure mortality ASR of 0.46 (95% CI = 0.31 to 0.75), Italy with an ASR of 0.82 (95% CI = 0.50 to 1.27) and Japan with an ASR of 0.26 (95% CI = 0.16 to 0.36).

## 4. Discussion

As indicated by the AAPI scores across different countries with different SDI scores, countries with high-middle SDI scores had the greatest improvement in disease burden and mortality associated with growth failure. In contrast, low-SDI countries had the highest mortality and disease burden rates in 1990 and 2021, with the lowest rate of improvement in disease burden and mortality as compared to countries with middle or high SDI scores. The high growth failure YLDs, DALYs, and mortality in low SDI regions in 2021 indicated that the impact of growth failure is highest amongst children residing in low-SDI countries.

The greatest improvement in growth failure DALYs, YLDs, and mortality was observed amongst children under 5 years of age across all SDI groups, regions, and countries. This is in line with global efforts to help achieve the proposed SDG to end preventable under-5 mortalities by 2030 [17]. Around 45% of under-5 mortalities are attributed to malnutrition-related causes, including growth failure as the main manifestation of malnutrition in children [4]. Despite significant improvements in the burden of growth failure in children under 5 years of age, current results from this study indicated that low SDI countries in Oceania and Sub-Saharan Africa are not on track to meet the 2025 target of a 40% reduction in stunting and wasting by 2025. No significant decrease in growth failure disease burden has been observed for both children under 5 years of age and for adolescents 15 to 19 years of age by 2021 in Oceania and Sub-Saharan Africa. Similar trends of insufficient progress have been identified in previous papers, indicating that the current annual rate of reduction (AARR) must be doubled to reach the global target of a 40% reduction in stunting by 2025 [18].

Furthermore, efforts need to be targeted towards low SDI countries in the Sub-Saharan African regions with a significant proportion of children affected by growth failure. Previous studies have estimated that children in Sub-Saharan Africa account for approximately one-third of all undernourished children globally, with around 39% stunted and 10% wasted [19]. The unequally high burden of growth failure and lower rates of improvement may be explained by the various SES disadvantages experienced by countries with low SDI scores. Factors including inadequate quality and access to health care services, food insecurity, and poor nutrition have been identified as predictors of growth failure in previous global studies [10,20]. Nutrition for young children is critical to support rapid development and to prevent premature mortality associated with malnutrition [13]. In contrast, chronic malnutrition in low SDI countries associated with rural residency, low parental education, lack of access to clean drinking water, and frequent episodes of diarrheal disease with poor treatment regimes all contribute to an increased risk of growth failure [1,19,20]. While low parental education can lead to poor nutritional knowledge and poor feeding practices, lack of access to clean drinking water can increase the risk of contracting diarrheal disease, further depleting nutritional stores and contributing to worse nutritional status [19,21]. As chronic malnutrition appears to be multi-factorial, comprehensive interventions to combat diarrheal disease and nutritional supplementation to maintain adequate nutritional status are required. To combat the burden of diarrheal disease, zinc and oral rehydration supplementation has been recommended in regions with endemic diarrheal disease [22]. Implementation of nutritional education programs for mothers has also significantly improved food and nutrient consumption and knowledge and dietary practices in complementary feeding in infants, indicating its efficacy in curbing infant malnutrition [23]. Despite the identification of evidence-based interventions, adequate coverage must be achieved to ensure the accessibility of such interventions for mothers and infants at risk of chronic malnutrition. Thus, to reach the 2025 nutritional targets, previous reviews have indicated the need to scale up current nutritional interventions, yielding an additional cost of $8.50 per child per year [18].

Significant differences in the extent of improvement in growth failure disease burden and mortality were also observed amongst different age groups. The significant improvements in under-5 growth failure disease burden and mortality may be attributed to global initiatives and national policies and interventions aimed at reducing maternal and early child nutrition [24,25]. One global review of national nutritional policy and intervention found a significant reduction in the prevalence of stunting associated with long-term protein and micronutrient supplementation lasting 3 to 7 years [25]. School feeding programs have also demonstrated significant success in improving stunting and wasting [26]. In addition to supplementation and feeding programs, maternal nutritional education, and counseling were also associated with significantly reduced prevalence of stunting in multiple countries including Bangladesh, Peru, and Sub-Saharan African countries [27,28].

Although substantial improvements in growth failure burden and mortalities have been achieved in children under 5, improvements in adolescents have been less pronounced. The result suggested that the improvement in adolescent growth failure DALYs is less than half of what has been achieved in the under-5 age group. Although growth failure in adolescents has received less attention in research, adolescence is a period of rapid physical and cognitive development with increased nutritional requirements [12,13]. Growth failure in adolescents has been linked to worse physical and cognitive outcomes, with negative economic consequences due to reduced work capacity [12]. The majority of research and global nutritional initiatives have been focused on younger children, which is reflected in the lower growth failure improvement rates observed amongst adolescents in this study [29]. However, just like younger children, adolescents with growth failure face significant risks of mortality and morbidity which should not be overlooked. Our study underscores the need for more research and targeted interventions for adolescents, stressing the importance of addressing growth failure during this crucial stage of development. Focusing on adolescents is vital not only for improving their immediate health but also for achieving long-term global health goals, like the SDGs, which aim to ensure healthy lives and well-being for all ages.

Although growth failure acts as an indicative measure of child undernutrition, malnutrition encompasses many aspects, including specific vitamin deficiencies and overnutrition that result in overweight and obesity [2]. One limitation of the current study was that only one aspect of malnutrition of growth failure was assessed. A growing number of evidence has indicated concerns for the double burden of nutrition where undernutrition-associated stunting and wasting co-exist with overnutrition associated with overweight and obesity [2]. While chronic undernutrition in LMICs contributes to a significant burden of growth failure and long-term cognitive and physical developmental problems, overnutrition manifested as overweight and obesity also has significant impacts on cardiovascular and metabolic diseases. Nonetheless, this study has comprehensively analyzed global trends of undernutrition in children and adolescents under 20 years of age, identifying countries and age groups of children that require further support to achieve the global nutritional targets.

## 5. Conclusions

Significant improvements in growth failure DALYs, YLDs, and mortality have been identified in children of all age groups across regions of all SDI levels from 1990 to 2021. The greatest improvements have been observed among children in the high-middle SDI group, while the least improvement was identified among children in the lowest SDI group. When children were divided into different age groups, growth failure DALYs, YLDs, and mortality decreased the most in children under 5 years of age from 1990 to 2021, while adolescents between ages 15 to 19 were the least improved. Although results from this study show promising trends in improving growth failure in younger children, adolescents with growth failure are similarly vulnerable to associated mortalities and morbidities and should not be neglected in policies and global initiatives. To reduce the national prevalence of growth failure a holistic approach including long-term nutritional supplementation and maternal nutritional education is required. With growing concerns about the double burden of nutrition, future studies evaluating child malnutrition should measure outcomes of under and overnutrition.

## Figures and Tables

**Table 1:** Under 20 years growth failure disease burden (DALYs and YLDs) and mortality from 1990 and 2021 across countries of high to low SDI scores.

		1990	2021	AAPC ^1^ (95% CI ^2^)	1990	2021	AAPC (95% CI)	1990	2021	AAPC (95% CI)	1990	2021	AAPC (95% CI)	1990	2021	AAPC (95% CI)	1990	2021	AAPC (95% CI)
		DALYs ^3^						Death						YLDs ^4^					
		Rate	Rate		Number	Number		Rate	Rate		Number	Number		Rate	Rate		Number	Number	
Global	<5	52,106.1	10,671.23	−0.79 (−0.85, −0.73)	329,371,060.29	70,235,044.84	−0.78 (−0.85, −0.71)	593.25	117.53	−0.8 (−0.86, −0.73)	3,750,014.12	773,565.49	−0.78 (−0.85, −0.72)	316.66	261.17	−0.53 (−0.54, −0.48)	2,001,631.68	1,718,930.99	−0.5 (−0.52, −0.44)
	5–9	370.17	62.88	−0.77 (−0.81, −0.71)	2,166,082.21	432,040.45	−0.72 (−0.78, −0.66)	4.21	0.61	−0.79 (−0.83, −0.74)	24,660.43	4214.18	−0.76 (−0.8, −0.69)	25.52	12.07	−0.48 (−0.53, −0.44)	149,357.22	82,923.98	−0.39 (−0.44, −0.34)
	10–14	97.66	35.81	−0.58 (−0.66, −0.51)	524,126.41	238,700.1	−0.48 (−0.57, −0.39)	1.02	0.32	−0.65 (−0.71, −0.57)	5476.69	2124.96	−0.56 (−0.64, −0.47)	19.62	11.09	−0.28 (−0.33, −0.22)	105,312.57	73,929.77	−0.1 (−0.17, −0.03)
	15–19	64.44	31.76	−0.46 (−0.54, −0.37)	334,824.51	1,981,65.57	−0.35 (−0.45, −0.25)	0.62	0.26	−0.57 (−0.65, −0.48)	3196.24	1623.24	−0.48 (−0.58, −0.37)	20.48	12.88	−0.14 (−0.2, −0.06)	106,428.77	80,397.87	0.04 (−0.04, 0.13)
High SDI ^5^	<5	935.46	112.8	−0.88 (−0.92, −0.85)	539,215.78	60,739.27	−0.9 (−0.93, −0.87)	10.55	1.1	−0.9 (−0.92, −0.87)	6083.37	590.93	−0.91 (−0.93, −0.88)	12	14.98	−0.67 (−0.94, −0.56)	6919.26	8066.39	−0.71 (−0.95, −0.62)
	5–9	25.02	11.26	0.38 (−0.2, 1.3)	14,482.11	6612.95	0.3 (−0.25, 1.16)	0.03	0.01	−0.72 (−0.79, −0.63)	16.81	7.78	−0.74 (−0.8, −0.65)	22.65	10.17	1.39 (0.1, 4.03)	13,107.68	5968.74	1.24 (0.03, 3.72)
	10–14	21.54	12.13	1 (0.1, 2.47)	12,310.01	7278.42	0.95 (0.07, 2.39)	0.02	0.01	−0.55 (−0.63, −0.46)	11.81	8.85	−0.56 (−0.64, −0.48)	19.96	10.99	2.13 (0.37, 6.25)	11,407.48	6593.32	2.05 (0.33, 6.08)
	15–19	22.94	15.62	1.42 (0.35, 3.09)	14,102.76	9403.81	1.22 (0.24, 2.76)	0.02	0.03	−0.32 (−0.42, −0.22)	12.41	16.64	−0.37 (−0.47, −0.28)	21.49	13.62	2.87 (0.65, 9.24)	13,216.24	8199.74	2.55 (0.52, 8.4)
High-middle SDI	<5	11,585.57	545.02	−0.94 (−0.96, −0.93)	12,107,630.28	381,755.92	−0.96 (−0.97, −0.95)	130.62	5.6	−0.95 (−0.96, −0.94)	136,510	3921.63	−0.96 (−0.97, −0.95)	120.61	46.31	−0.65 (−0.69, −0.48)	126,049.91	32,440.07	−0.73 (−0.77, −0.61)
	5–9	65.09	7.49	−0.79 (−0.83, −0.73)	65,637.79	6175.54	−0.81 (−0.85, −0.75)	0.53	0.05	−0.85 (−0.88, −0.82)	529.83	42.3	−0.87 (−0.89, −0.84)	22.13	3.24	−0.46 (−0.61, −0.25)	22,316.07	2672.87	−0.52 (−0.65, −0.32)
	10–14	31.53	6.82	−0.65 (−0.72, −0.56)	30,964.46	5346.77	−0.7 (−0.75, −0.61)	0.18	0.04	−0.77 (−0.81, −0.73)	173.44	35.09	−0.8 (−0.83, −0.76)	18.03	3.35	−0.24 (−0.44, 0.1)	17,708.95	2629.39	−0.34 (−0.51, −0.04)
	15–19	30.97	7.41	−0.54 (−0.63, −0.4)	31,503.98	5371.4	−0.66 (−0.73, −0.55)	0.17	0.05	−0.72 (−0.76, −0.68)	169.38	33.33	−0.79 (−0.82, −0.76)	19.08	4.08	−0.03 (−0.29, 0.44)	19,403.99	2955.78	−0.27 (−0.46, 0.08)
Middle SDI	<5	30,607	3183.23	−0.89 (−0.92, −0.86)	62,693,093.94	5,622,138.13	−0.9 (−0.93, −0.88)	346.75	33.86	−0.9 (−0.92, −0.87)	710,261.66	59,801.36	−0.91 (−0.93, −0.88)	223.41	171.04	−0.57 (−0.61, −0.44)	457,609.46	302,081.92	−0.62 (−0.66, −0.51)
	5–9	151.34	31.28	−0.75 (−0.79, −0.7)	293,500.23	61,648.25	−0.74 (−0.78, −0.7)	1.58	0.25	−0.8 (−0.84, −0.77)	3073.69	497.39	−0.8 (−0.83, −0.77)	21.74	10.38	−0.41 (−0.46, −0.35)	42,164.61	20,465.71	−0.39 (−0.45, −0.34)
	10–14	55.9	23.59	−0.6 (−0.65, −0.54)	102,078.45	45,575.22	−0.57 (−0.63, −0.52)	0.5	0.17	−0.7 (−0.74, −0.66)	905.29	337.25	−0.69 (−0.72, −0.65)	18	10.07	−0.23 (−0.29, −0.15)	32,875.8	19,443.9	−0.19 (−0.25, −0.11)
	15–19	43.88	22.05	−0.47 (−0.54, −0.4)	81,435.85	40,211.04	−0.48 (−0.55, −0.41)	0.35	0.14	−0.65 (−0.69, −0.61)	646.03	250.84	−0.66 (−0.7, −0.62)	19.01	12.09	−0.06 (−0.14, 0.04)	35,285.69	22,038.08	−0.08 (−0.16, 0.01)
Low-middle SDI	<5	76,429.45	9779.82	−0.86 (−0.9, −0.82)	128,558,612.44	18,735,979.88	−0.85 (−0.89, −0.81)	869.34	106.28	−0.87 (−0.9, −0.83)	1462,275.01	203,607.28	−0.85 (−0.89, −0.81)	546.5	336.99	−0.62 (−0.64, −0.56)	919,238.61	645,595.51	−0.58 (−0.6, −0.52)
	5–9	808.78	62.61	−0.88 (−0.91, −0.83)	1,233,154.42	122,002.92	−0.85 (−0.89, −0.79)	9.49	0.55	−0.9 (−0.93, −0.86)	14,469.39	1064.26	−0.88 (−0.91, −0.83)	32.65	17.37	−0.58 (−0.63, −0.54)	49,776.66	33,855.74	−0.49 (−0.55, −0.44)
	10–14	154.93	37.28	−0.69 (−0.76, −0.61)	205,621.08	72,090.77	−0.57 (−0.66, −0.46)	1.72	0.28	−0.77 (−0.82, −0.69)	2287.87	537.77	−0.68 (−0.75, −0.57)	23.1	15.72	−0.42 (−0.48, −0.36)	30,655.8	30,399.46	−0.19 (−0.27, −0.11)
	15–19	100.17	31.91	−0.62 (−0.69, −0.54)	117,398.19	58,884.36	−0.41 (−0.51, −0.29)	1.07	0.2	−0.74 (−0.8, −0.65)	1256.24	364.6	−0.59 (−0.69, −0.46)	23.57	17.57	−0.39 (−0.45, −0.33)	27,625.39	32,430.89	−0.05 (−0.14, 0.04)
Low SDI	<5	129,754.74	27,408.96	−0.8 (−0.87, −0.74)	125,311,062.81	45,382,461.21	−0.64 (−0.76, −0.53)	1483.86	305.04	−0.8 (−0.87, −0.74)	1,433,045.77	505,070	−0.64 (−0.76, −0.53)	508.49	440.78	−0.57 (−0.59, −0.52)	491,077.48	729,825.13	−0.21 (−0.25, −0.13)
	5–9	700.34	153.29	−0.75 (−0.81, −0.68)	558,352.36	235,287.21	−0.5 (−0.61, −0.35)	8.23	1.69	−0.76 (−0.82, −0.68)	6559.72	2598.91	−0.51 (−0.63, −0.36)	27.51	12.99	−0.63 (−0.67, −0.58)	21,934.05	19,939.95	−0.25 (−0.34, −0.14)
	10–14	262.98	76.69	−0.71 (−0.77, −0.65)	172,818.49	108,253.6	−0.35 (−0.48, −0.2)	3.19	0.85	−0.73 (−0.79, −0.66)	2094.51	1204.22	−0.38 (−0.52, −0.22)	19.2	10.52	−0.56 (−0.62, −0.49)	126,18.91	14,845.54	0 (−0.13, 0.15)
	15–19	169.02	67.89	−0.68 (−0.75, −0.6)	90,180.36	84,172.79	−0.22 (−0.38, −0.02)	2.08	0.77	−0.7 (−0.77, −0.61)	1110.04	956.45	−0.27 (−0.44, −0.05)	20.33	11.9	−0.53 (−0.59, −0.46)	10,847.12	14,751.66	0.14 (−0.01, 0.31)

^1^ Average annual percentage change; ^2^ Confidence Interval; ^3^ Disability adjusted life years; ^4^ Years loss to disability; ^5^ Sociodemographic index.

**Table 2:** Under 5 growth failure DALYs, mortality, YLDs across 21 geographical regions.

	DALYs ^1^						Death						YLDs ^2^					
	1990	2021	AAPC (95% CI ^3^)	1990	2021	AAPC	1990	2021	AAPC (95% CI)	1990	2021	AAPC	1990	2021	AAPC (95% CI)	1990	2021	AAPC (95% CI)
	Rate	Rate		Number	Number		Rate	Rate		Number	Number		Rate	Rate		Number	Number	
Andean Latin America	31,617.51	2653.08	−0.93 (−0.95, −0.91)	1,722,076.11	163,318.29	−0.92 (−0.94, −0.89)	361.49	29.63	−0.93 (−0.95, −0.91)	19,688.95	1823.79	−0.92 (−0.94, −0.89)	11.27	17.63	−0.88 (−0.93, −0.85)	613.62	1085.15	−0.86 (−0.91, −0.83)
Australasia	310.42	33.8	−0.85 (−0.89, −0.81)	4789.24	613.76	−0.82 (−0.87, −0.78)	3.41	0.35	−0.86 (−0.89, −0.83)	52.57	6.31	−0.84 (−0.87, −0.8)	10.61	2.8	−0.22 (−0.39, −0.1)	163.64	50.84	−0.09 (−0.28, 0.06)
Caribbean	37,134.73	11,618.89	−0.69 (−0.77, −0.59)	1,537,042.03	449,439.7	−0.71 (−0.78, −0.62)	423.27	129.74	−0.69 (−0.77, −0.59)	17,519.55	5018.69	−0.71 (−0.78, −0.62)	64.17	67.8	−0.49 (−0.57, −0.16)	2656.24	2622.44	−0.52 (−0.6, −0.21)
Central Asia	36,640.99	5049.24	−0.86 (−0.89, −0.83)	3,473,308.19	504,773.55	−0.85 (−0.88, −0.82)	416.1	56.21	−0.86 (−0.89, −0.83)	39,443.33	5619.39	−0.85 (−0.88, −0.82)	182.46	46.45	−0.8 (−0.83, −0.75)	17,296.02	4643.24	−0.79 (−0.82, −0.73)
Central Europe	5301.53	509.09	−0.9 (−0.92, −0.88)	473,621.65	28,435.98	−0.94 (−0.95, −0.93)	59.67	5.6	−0.9 (−0.92, −0.88)	5330.31	312.89	−0.94 (−0.95, −0.93)	53.65	8.98	−0.81 (−0.85, −0.77)	4792.86	501.63	−0.88 (−0.91, −0.86)
Central Latin America	21,433.29	2573.13	−0.89 (−0.92, −0.85)	4,907,021.29	516,951.7	−0.9 (−0.93, −0.87)	243.99	28.7	−0.89 (−0.92, −0.85)	55,859.12	5766.48	−0.9 (−0.93, −0.87)	36.98	16.92	−0.83 (−0.95, −0.78)	8467.11	3399.75	−0.85 (−0.96, −0.81)
Central Sub-Saharan Africa	109,384.81	17,648.65	−0.86 (−0.95, −0.79)	11,758,826.6	3,717,953.14	−0.72 (−0.91, −0.58)	1250.3	196	−0.86 (−0.95, −0.8)	134,406.58	41,289.87	−0.72 (−0.91, −0.59)	308.77	324.91	−0.61 (−0.69, −0.55)	33,192.21	68,447.07	−0.21 (−0.38, −0.09)
East Asia	26,230.67	594.16	−0.97 (−0.98, −0.96)	31,433,108.52	475,768.01	−0.98 (−0.99, −0.97)	297.91	6.6	−0.97 (−0.98, −0.96)	356,998.01	5286.23	−0.98 (−0.99, −0.97)	48.33	5.17	−0.96 (−1, −0.94)	57,916.39	4136.15	−0.97 (−1, −0.96)
Eastern Europe	2728.77	404.18	−0.87 (−0.88, −0.85)	470,123.83	40,899.14	−0.92 (−0.93, −0.91)	30.25	3.93	−0.88 (−0.9, −0.86)	5210.86	397.73	−0.93 (−0.94, −0.92)	75.13	53.24	−0.66 (−0.73, −0.53)	12,943.29	5387.4	−0.8 (−0.84, −0.72)
Eastern Sub-Saharan Africa	129,209.82	20,581.57	−0.85 (−0.89, −0.8)	46,541,253.21	13,130,295.35	−0.73 (−0.81, −0.64)	1479.19	229.67	−0.85 (−0.89, −0.8)	532,802.01	146,523.04	−0.73 (−0.81, −0.64)	269.92	260.86	−0.61 (−0.65, −0.25)	97,223.27	166,416.92	−0.32 (−0.38, 0.32)
High-income Asia Pacific	507.45	105	−0.83 (−0.91, −0.78)	52,017.87	6774.72	−0.89 (−0.94, −0.86)	5.65	0.87	−0.87 (−0.89, −0.84)	579.48	56.05	−0.92 (−0.93, −0.9)	12.78	27.74	−0.2 (−0.64, 0.08)	1309.79	1789.88	−0.49 (−0.77, −0.32)
High-income North America	235.79	83.21	−0.72 (−0.76, −0.68)	50,688.99	17,056.46	−0.74 (−0.77, −0.7)	2.69	0.92	−0.72 (−0.76, −0.68)	577.24	189.47	−0.74 (−0.77, −0.7)	0	0.75	−0.9 (−0.93, −0.87)	0.35	153.71	−0.9 (−0.94, −0.88)
North Africa and Middle East	39,237.31	3684.53	−0.89 (−0.92, −0.86)	20,929,881.33	2,252,612.09	−0.87 (−0.9, −0.84)	445.6	39.81	−0.89 (−0.92, −0.87)	237,692.96	24,339.78	−0.87 (−0.9, −0.84)	244.31	144.26	−0.66 (−0.69, −0.54)	130,319.45	88,195.22	−0.6 (−0.63, −0.46)
Oceania	44,750.74	21,627.88	−0.57 (−0.68, −0.45)	439,974.04	418,380.72	−0.18 (−0.38, 0.06)	508.26	237.91	−0.58 (−0.69, −0.45)	4997.04	4602.19	−0.19 (−0.4, 0.05)	349.46	449.44	−0.06 (−0.54, 1.46)	3435.8	8694.27	0.82 (−0.11, 3.73)
	26.35	19.83	−0.33 (−0.52, −0.04)	179.85	258.83	0.27 (−0.09, 0.82)	0.23	0.14	−0.34 (−0.6, 0.06)	1.56	1.79	0.25 (−0.23, 1.02)	10.05	9.86	−0.32 (−0.56, 0.06)	68.6	128.71	0.3 (−0.16, 1.02)
South Asia	69,460.79	8918.68	−0.87 (−0.9, −0.84)	112,367,622.09	14,144,465.87	−0.87 (−0.9, −0.83)	787.69	94.14	−0.88 (−0.91, −0.84)	1,274,251.28	149,303.18	−0.88 (−0.91, −0.84)	784.29	536.56	−0.55 (−0.57, −0.48)	1,268,748.12	850,953.58	−0.55 (−0.57, −0.48)
Southeast Asia	45,115.5	4669.09	−0.9 (−0.92, −0.87)	26,836,444.14	2,627,977.97	−0.9 (−0.92, −0.87)	511.53	49.6	−0.9 (−0.92, −0.87)	304,277.94	27,918.94	−0.91 (−0.93, −0.88)	373.07	256.61	−0.62 (−0.64, −0.54)	221,918.42	144,434.14	−0.63 (−0.65, −0.56)
Southern Latin America	4344.73	376.67	−0.91 (−0.93, −0.89)	224,155.21	16,115.49	−0.93 (−0.95, −0.91)	49.28	4.11	−0.92 (−0.93, −0.89)	2542.28	176.03	−0.93 (−0.95, −0.91)	11.62	10.38	−0.69 (−0.84, −0.59)	599.6	444.26	−0.74 (−0.87, −0.66)
Southern Sub-Saharan Africa	44,130.73	16,117.96	−0.66 (−0.73, −0.58)	3,165,902.4	1294,129.36	−0.63 (−0.71, −0.54)	502.33	180.4	−0.66 (−0.73, −0.58)	36,036.7	14,484.17	−0.63 (−0.71, −0.55)	147.37	67.31	−0.72 (−0.75, −0.69)	10,572.39	5404.78	−0.7 (−0.73, −0.67)
Tropical Latin America	30,156.93	942.44	−0.96 (−0.97, −0.95)	5,294,119.65	162,171.17	−0.96 (−0.97, −0.95)	342.21	10.45	−0.96 (−0.97, −0.95)	60,075.16	1797.36	−0.96 (−0.97, −0.95)	41.71	11.5	−0.88 (−1.03, −0.77)	7321.95	1979.52	−0.88 (−1.03, −0.77)
Western Europe	273.06	59.58	−0.78 (−0.9, −0.72)	62,692.63	12,647.51	−0.8 (−0.91, −0.75)	3.1	0.5	−0.82 (−0.86, −0.79)	710.97	106.24	−0.83 (−0.87, −0.81)	1.36	14.94	−0.22 (−1.81, 0.66)	313.32	3170.75	−0.28 (−1.75, 0.54)
Western Sub-Saharan Africa	161,540.54	37,837.65	−0.76 (−0.85, −0.69)	57,626,391.26	30,254,264.87	−0.47 (−0.67, −0.31)	1852.83	423.41	−0.76 (−0.85, −0.69)	660,961.77	338,547.65	−0.47 (−0.67, −0.31)	341.51	446.51	−0.53 (−0.58, −0.33)	121,827.84	357,020.29	0.06 (−0.05, 0.5)

^1^ Disability adjusted life years; ^2^ Years loss to disability; ^3^ Confidence Interval.

**Table 3:** 15 to 19 years old growth failure DALYs, mortalities and YLDs in 1990 and 2021 in 21 geographical regions.

	DALYs ^1^						Death						YLDs ^2^					
	1990	2021	AAPC (95% CI ^3^)	1990	2021	AAPC (95% CI)	1990	2021	AAPC (95% CI)	1990	2021	AAPC (95% CI)	1990	2021	AAPC (95% CI)	1990	2021	AAPC (95% CI)
	Rate	Rate		Number	Number		Rate	Rate		Number	Number		Rate	Rate		Number	Number	
Andean Latin America	76.07	27.27	−0.67 (−0.76, −0.55)	3112.29	1521.34	−0.55 (−0.67, −0.39)	0.98	0.31	−0.72 (−0.79, −0.63)	40.21	17.56	−0.62 (−0.72, −0.49)	5.86	4.45	-	239.92	248.28	-
Australasia	10.47	0.86	0.15 (−0.43, 4.26)	176.73	15.48	0.23 (−0.39, 4.64)	0.01	0.01	−0.24 (−0.43, 0)	0.14	0.14	−0.19 (−0.39, 0.07)	9.87	0.3	-	166.64	5.38	-
Caribbean	62.15	24.32	−0.4 (−0.56, −0.18)	2283.96	913.83	−0.38 (−0.55, −0.16)	0.7	0.32	−0.39 (−0.55, −0.16)	25.67	12.19	−0.37 (−0.55, −0.14)	12.27	0.81	−0.58 (−0.85, −0.17)	451.04	30.62	−0.58 (−0.84, −0.15)
Central Asia	13.44	2.85	−0.75 (−0.8, −0.68)	885.12	197.89	−0.73 (−0.79, −0.66)	0.07	0.02	−0.7 (−0.75, −0.64)	4.5	1.49	−0.68 (−0.73, −0.62)	8.57	1.29	−0.79 (−0.87, −0.66)	564.12	89.88	−0.78 (−0.87, −0.65)
Central Europe	15.37	2.61	0.12 (−0.15, 0.44)	1478.05	152.95	−0.33 (−0.49, −0.14)	0.01	0.03	0.93 (0.67, 1.2)	1.38	1.92	0.16 (0, 0.31)	14.35	0.24	−0.79 (−0.91, −0.61)	1379.58	13.83	−0.87 (−0.94, −0.77)
Central Latin America	86.1	36.54	−0.59 (−0.64, −0.55)	15,665.47	7965.66	−0.51 (−0.57, −0.47)	0.98	0.48	−0.6 (−0.64, −0.56)	178.98	104.17	−0.52 (−0.57, −0.47)	15.86	1.94	−0.41 (−0.65, −0.04)	2885.79	422.45	−0.3 (−0.58, 0.15)
Central Sub-Saharan Africa	151.11	63.9	−0.6 (−0.73, −0.4)	8589.58	9507.89	0.06 (−0.3, 0.57)	2.01	0.84	−0.59 (−0.73, −0.38)	114.21	124.6	0.07 (−0.3, 0.63)	7.51	3.11	−0.69 (−0.83, −0.48)	426.82	462.95	−0.19 (−0.56, 0.35)
East Asia	31.78	3.43	−0.86 (−0.89, −0.82)	41,477.77	2662.38	−0.92 (−0.94, −0.89)	0.24	0.04	−0.85 (−0.89, −0.81)	309.96	32.9	−0.91 (−0.93, −0.89)	14.81	0.36	−0.9 (−0.98, −0.7)	19,324.84	280.03	−0.94 (−0.99, −0.82)
Eastern Europe	21.13	6.21	−0.53 (−0.63, −0.38)	3352.41	665.21	−0.68 (−0.75, −0.58)	0.05	0.03	−0.47 (−0.54, −0.39)	8.5	3.16	−0.64 (−0.69, −0.58)	17.31	4.07	−0.55 (−0.7, −0.33)	2745.74	436.43	−0.7 (−0.79, −0.55)
Eastern Sub-Saharan Africa	299.57	110.35	−0.72 (−0.79, −0.64)	60,705.53	54,239.83	−0.33 (−0.49, −0.13)	4.02	1.47	−0.72 (−0.79, −0.63)	813.82	722.61	−0.32 (−0.49, −0.11)	12.54	3.65	−0.78 (−0.81, −0.73)	2541.39	1793.99	−0.46 (−0.55, −0.35)
High-income Asia Pacific	12.58	1.69	−0.51 (−0.66, −0.29)	1904.28	141.41	−0.73 (−0.81, −0.61)	0.02	0.02	−0.49 (−0.6, −0.31)	2.45	1.86	−0.72 (−0.78, −0.62)	11.42	0.08	−0.76 (−1, 2.02)	1729.44	6.9	−0.87 (−1, 0.67)
High-income North America	16.59	27.71	2.37 (0.18, 16.34)	3324.06	6634.58	3.03 (0.41, 19.71)	0.01	0.04	0.96 (0.86, 1.08)	1.89	9.16	1.34 (1.22, 1.48)	15.91	24.95	2.67 (0.13, 256.9)	3189.28	5971.75	3.38 (0.34, 306.89)
North Africa and Middle East	31.8	14.84	−0.61 (−0.69, −0.51)	11,630.63	7890.88	−0.44 (−0.55, −0.28)	0.28	0.13	−0.59 (−0.71, −0.44)	100.69	71.77	−0.4 (−0.57, −0.17)	12.12	5.04	−0.65 (−0.73, −0.48)	4434.09	2681.65	−0.49 (−0.6, −0.24)
Oceania	26.35	19.83	−0.33 (−0.52, −0.04)	179.85	258.83	0.27 (−0.09, 0.82)	0.23	0.14	−0.34 (−0.6, 0.06)	1.56	1.79	0.25 (−0.23, 1.02)	10.05	9.86	−0.32 (−0.56, 0.06)	68.6	128.71	0.3 (−0.16, 1.02)
South Asia	96.16	36.57	−0.54 (−0.65, −0.42)	104,890.26	64,544.25	−0.26 (−0.44, −0.06)	0.87	0.06	−0.89 (−0.93, −0.82)	944.72	107.53	−0.81 (−0.88, −0.7)	34.27	32.15	−0.21 (−0.27, −0.14)	37,376.2	56,741.39	0.27 (0.18, 0.38)
Southeast Asia	84.92	26.03	−0.61 (−0.68, −0.55)	41,660.04	14,738.86	−0.55 (−0.63, −0.48)	0.81	0.21	−0.66 (−0.74, −0.55)	397.33	116.88	−0.61 (−0.71, −0.49)	27.06	11.07	−0.53 (−0.58, −0.45)	13,274.97	6264.98	−0.45 (−0.52, −0.37)
Southern Latin America	16.47	9.75	−0.35 (−0.55, 0)	732.01	488.68	−0.27 (−0.49, 0.13)	0.14	0.11	−0.48 (−0.56, −0.39)	6.28	5.37	−0.42 (−0.5, −0.31)	6.38	1.98	-	283.71	99.44	-
Southern Sub-Saharan Africa	50	34.79	−0.02 (−0.28, 0.34)	2916.09	2504.22	0.22 (−0.1, 0.67)	0.49	0.47	0.11 (−0.19, 0.52)	28.62	33.76	0.38 (0.01, 0.9)	14.99	0.78	−0.84 (−0.89, −0.78)	874.11	56.25	−0.8 (−0.86, −0.72)
Tropical Latin America	32.4	17.82	−0.34 (−0.41, −0.25)	5083.28	2920.77	−0.31 (−0.38, −0.22)	0.3	0.22	−0.32 (−0.38, −0.26)	47.83	35.71	−0.28 (−0.35, −0.22)	10.63	2.05	−0.47 (−0.7, 0.13)	1668.32	336.37	−0.44 (−0.69, 0.19)
Western Europe	39.69	9.17	6.93 (0.91, 24.36)	10,855.22	2163.2	5.85 (0.65, 20.89)	0.01	0.01	−0.24 (−0.34, −0.12)	2.28	1.94	−0.35 (−0.43, −0.24)	39.09	8.58	-	10,692.52	2023.11	-
Western Sub-Saharan Africa	71.31	33.52	−0.58 (−0.66, −0.49)	13,921.88	18,037.43	0.15 (−0.07, 0.41)	0.85	0.4	−0.56 (−0.66, −0.44)	165.24	216.73	0.21 (−0.06, 0.54)	10.82	4.28	−0.69 (−0.74, −0.64)	2111.66	2303.46	−0.16 (−0.29, −0.01)

^1^ Disability-adjusted life years; ^2^ Years loss to disability; ^3^ Confidence Interval.

## Data Availability

Data analyzed will be made available upon request.

## Supplementary Materials

The following supporting information can be downloaded at: https://www.mdpi.com/article/10.3390/nu17071185/s1, Table S1: National under 20 growth failure DALYs, mortalities and YLDs in 1990 and 2021 in 204 countries.

## Abbreviations

The following abbreviations are used in this manuscript:

LMICs	Low to middle income countries
SDG	Sustainable developmental goals
WHO	World Health Organization
DALYs	Disability Adjusted Life Years
YLDs	Years Loss to Disability
AAPC	Average Annual Percentage Change
ASR	Age-standardized rate
SDI	Sociodemographic index score
LDI	Lag distributed income
CI	Confidence interval
