# Supplementary material for: Global, Regional, and National Burden of Child Growth Failure, 1990–2021: A Systematic Analysis for the Global Burden of Disease Study 2021
Source: Nutrients. 2025 Mar 28;17(7):1185. doi: 10.3390/nu17071185 (PMC11990353; doi:10.3390/nu17071185)
Supplement: Supplementary file 1 [file nutrients-17-01185-s001.zip › nutrients-3521280-supplementary.pdf]

Supplementary Table S1: National under 20 growth failure DALYs, mortalities and YLDs in 1990 and 2021 in 204 countries

	DALYs		Mortalities		YLDs	
	1990	2021	1990	2021	1990	2021
**Afghanistan**	28770.24 (22485.22, 37271.30)	3989.87 (2770.56, 5364.68)	322.88 (251.53, 418.63)	44.48 (30.87, 59.86)	322.88 (251.53, 418.63)	40.53 (21.83, 64.21)
**Albania**	6085.22 (4929.25, 7229.48)	377.06 (258.29, 526.95)	66.49 (53.32, 79.27)	4.07 (2.73, 5.63)	66.49 (53.32, 79.27)	16.08 (7.45, 28.75)
**Algeria**	3466.30 (2527.11, 4709.69)	215.84 (139.28, 311.34)	37.48 (26.75, 51.18)	2.24 (1.40, 3.27)	37.48 (26.75, 51.18)	16.66 (8.81, 27.29)
**American Samoa**	1518.79 (1047.70, 2128.56)	715.41 (458.29, 1091.11)	17.05 (11.82, 23.84)	8.02 (5.16, 12.23)	17.05 (11.82, 23.84)	4.96 (-0.98, 12.84)
**Andorra**	62.03 (31.19, 113.66)	14.30 (0.10, 46.04)	0.66 (0.35, 1.20)	0.03 (0.01, 0.06)	0.66 (0.35, 1.20)	11.74 (-1.25, 41.10)
**Angola**	49257.26 (37165.58, 59349.97)	4055.43 (1426.52, 7020.06)	555.48 (419.54, 669.77)	45.47 (16.33, 78.53)	555.48 (419.54, 669.77)	42.27 (-12.06, 103.42)
**Antigua and Barbuda**	660.86 (476.88, 869.45)	338.69 (235.59, 456.18)	6.79 (4.74, 9.01)	3.75 (2.63, 5.06)	6.79 (4.74, 9.01)	7.35 (2.67, 14.55)
**Argentina**	1300.82 (995.01, 1580.35)	118.91 (75.37, 174.35)	14.48 (11.25, 17.50)	1.33 (0.87, 1.94)	14.48 (11.25, 17.50)	1.27 (-0.85, 3.82)
**Armenia**	4365.65 (2926.63, 5735.74)	545.60 (364.78, 781.08)	48.74 (32.82, 63.95)	6.04 (4.04, 8.65)	48.74 (32.82, 63.95)	6.96 (2.63, 12.98)
**Australia**	41.78 (26.54, 68.34)	6.51 (3.70, 11.35)	0.46 (0.31, 0.75)	0.07 (0.04, 0.12)	0.46 (0.31, 0.75)	0.30 (-0.12, 0.59)
**Austria**	49.01 (20.06, 85.12)	12.54 (1.31, 59.17)	0.45 (0.25, 0.71)	0.06 (0.03, 0.10)	0.45 (0.25, 0.71)	7.49 (-1.00, 51.20)
**Azerbaijan**	13418.63 (9883.11, 16275.12)	1606.69 (1072.92, 2224.30)	149.73 (110.44, 181.37)	17.75 (11.82, 24.69)	149.73 (110.44, 181.37)	26.50 (14.44, 41.86)
**Bahamas**	969.47 (637.48, 1285.43)	234.50 (143.71, 356.34)	10.85 (7.17, 14.38)	2.62 (1.61, 3.98)	10.85 (7.17, 14.38)	1.99 (0.22, 4.33)
**Bahrain**	951.88 (647.18, 1263.24)	93.10 (55.38, 136.76)	10.05 (6.74, 13.40)	1.02 (0.62, 1.49)	10.05 (6.74, 13.40)	2.95 (0.05, 8.77)
**Bangladesh**	21267.60 (17123.35, 24835.58)	1292.44 (884.90, 1731.68)	237.35 (190.29, 277.52)	13.71 (9.23, 18.70)	237.35 (190.29, 277.52)	78.20 (49.69, 113.90)
**Barbados**	593.82 (423.30, 765.00)	166.35 (101.37, 245.24)	6.47 (4.59, 8.34)	1.80 (1.09, 2.66)	6.47 (4.59, 8.34)	6.48 (2.17, 12.98)
**Belarus**	486.03 (299.43, 679.11)	57.57 (34.63, 89.32)	5.37 (3.38, 7.45)	0.62 (0.39, 0.96)	5.37 (3.38, 7.45)	2.22 (-0.60, 5.94)
**Belgium**	85.58 (45.06, 135.33)	30.58 (10.14, 72.71)	0.91 (0.51, 1.42)	0.27 (0.12, 0.49)	0.91 (0.51, 1.42)	6.27 (-0.78, 30.18)
**Belize**	4077.95 (2998.31, 5021.87)	581.39 (412.90, 795.42)	45.55 (33.66, 55.93)	6.50 (4.65, 8.89)	45.55 (33.66, 55.93)	5.75 (2.28, 10.57)
**Benin**	34380.79 (23313.67, 43833.67)	6534.07 (1114.82, 11690.61)	387.29 (263.51, 493.62)	73.09 (12.49, 131.21)	387.29 (263.51, 493.62)	89.33 (-9.29, 191.55)
**Bermuda**	224.27 (133.08, 334.33)	51.66 (27.78, 88.46)	2.49 (1.52, 3.69)	0.56 (0.32, 0.94)	2.49 (1.52, 3.69)	2.36 (-1.01, 6.76)
**Bhutan**	19744.51 (6847.02, 27059.31)	1244.10 (753.43, 1856.55)	221.11 (76.89, 302.78)	13.88 (8.40, 20.77)	221.11 (76.89, 302.78)	11.77 (6.26, 19.96)
**Bolivia (Plurinational State of)**	17379.66 (12665.01, 21636.62)	1387.06 (919.73, 1907.63)	195.44 (142.79, 243.19)	15.63 (10.40, 21.47)	195.44 (142.79, 243.19)	2.82 (-0.81, 7.24)
**Bosnia and Herzegovina**	224.49 (142.81, 306.33)	57.05 (32.53, 85.75)	2.36 (1.49, 3.24)	0.60 (0.34, 0.90)	2.36 (1.49, 3.24)	3.89 (1.84, 6.59)
**Botswana**	9716.94 (7092.56, 12055.64)	4295.22 (2708.97, 6080.88)	108.55 (79.45, 135.16)	48.10 (30.39, 68.19)	108.55 (79.45, 135.16)	33.19 (17.03, 53.94)
**Brazil**	6601.27 (4337.81, 8190.82)	250.80 (162.36, 354.44)	73.65 (48.66, 91.34)	2.80 (1.83, 3.93)	73.65 (48.66, 91.34)	3.42 (-0.41, 8.53)

**Brunei Darussalam**	238.06 (160.95, 309.67)	89.60 (54.67, 130.93)	2.57 (1.75, 3.36)	0.97 (0.60, 1.44)	2.57 (1.75, 3.36)	2.93 (0.82, 5.84)
**Bulgaria**	1124.10 (757.95, 1624.31)	192.93 (110.27, 296.15)	12.49 (8.40, 18.02)	2.10 (1.18, 3.26)	12.49 (8.40, 18.02)	5.78 (3.29, 9.14)
**Burkina Faso**	46553.29 (28240.23, 58727.56)	11505.01 (3773.39, 18625.31)	522.71 (319.24, 659.55)	128.45 (41.99, 208.82)	522.71 (319.24, 659.55)	154.84 (32.26, 287.03)
**Burundi**	37577.76 (14555.76, 53692.99)	6928.57 (-143.35, 13031.26)	424.47 (165.07, 606.13)	77.90	424.47 (165.07, 606.13)	64.18 (-28.12, 156.39)
**Cabo Verde**	10762.72 (8379.08, 12963.39)	554.42 (337.17, 840.83)	120.63 (94.34, 145.13)	6.23 (3.81, 9.44)	120.63 (94.34, 145.13)	1.81 (-1.16, 5.24)
**Cambodia**	24833.33 (18720.99, 30761.05)	2056.09 (1456.50, 2753.86)	276.81 (209.17, 343.45)	22.59 (16.09, 30.29)	276.81 (209.17, 343.45)	48.11 (26.93, 75.01)
**Cameroon**	21356.05 (12604.65, 28239.46)	5084.83 (1545.23, 8823.02)	240.64 (142.66, 318.09)	57.12 (17.47, 99.52)	240.64 (142.66, 318.09)	42.98 (-4.81, 103.75)
**Canada**	52.70 (28.53, 83.71)	34.58 (7.20, 69.45)	0.56 (0.33, 0.87)	0.17 (0.08, 0.29)	0.56 (0.33, 0.87)	19.06 (-0.21, 44.40)
**Central African Republic**	35316.97 (23205.30, 45093.22)	13125.81 (5931.03, 19882.69)	396.50 (261.96, 506.35)	147.02 (66.66, 223.47)	396.50 (261.96, 506.35)	128.11 (14.75, 262.06)
**Chad**	50415.14 (35258.64, 62521.74)	19225.92 (10921.40, 26348.53)	567.68 (397.67, 703.74)	215.75 (122.63, 296.40)	567.68 (397.67, 703.74)	163.06 (80.32, 252.77)
**Chile**	855.99 (572.69, 1197.16)	53.90 (34.98, 85.45)	9.56 (6.46, 13.33)	0.56 (0.40, 0.79)	9.56 (6.46, 13.33)	4.82 (-0.39, 17.02)
**China**	5798.39 (4212.31, 7102.90)	154.12 (101.15, 230.26)	64.87 (47.33, 79.52)	1.72 (1.14, 2.56)	64.87 (47.33, 79.52)	1.38 (0.05, 3.20)
**Colombia**	3065.77 (2183.08, 3954.84)	423.33 (265.12, 641.78)	34.36 (24.67, 44.21)	4.76 (3.02, 7.18)	34.36 (24.67, 44.21)	2.44 (-1.71, 7.66)
**Comoros**	22308.61 (16736.15, 26969.34)	3498.09 (2448.09, 4636.83)	249.68 (186.84, 302.65)	38.67 (26.77, 51.44)	249.68 (186.84, 302.65)	83.62 (51.93, 123.97)
**Congo**	14478.86 (7227.91, 20855.48)	2391.71 (700.71, 4265.43)	162.12 (81.24, 232.95)	26.45 (7.81, 46.94)	162.12 (81.24, 232.95)	54.95 (3.33, 117.35)
**Cook Islands**	1565.15 (907.12, 2445.99)	282.79 (148.04, 496.65)	17.54 (10.23, 27.38)	3.15 (1.66, 5.50)	17.54 (10.23, 27.38)	3.55 (-2.20, 10.82)
**Costa Rica**	738.94 (409.05, 1070.74)	113.59 (44.25, 197.92)	8.21 (4.63, 11.78)	0.93 (0.51, 1.58)	8.21 (4.63, 11.78)	31.35 (-1.34, 57.72)
**Croatia**	247.33 (171.99, 328.30)	79.06 (42.38, 120.54)	2.73 (1.91, 3.62)	0.87 (0.47, 1.32)	2.73 (1.91, 3.62)	1.62 (0.48, 3.16)
**Cuba**	490.05 (301.61, 677.46)	111.58 (67.34, 171.41)	5.46 (3.40, 7.51)	1.23 (0.76, 1.88)	5.46 (3.40, 7.51)	1.85 (-0.85, 5.20)
**Cyprus**	142.13 (64.52, 226.61)	16.07 (2.94, 31.72)	1.51 (0.72, 2.40)	0.14 (0.05, 0.26)	1.51 (0.72, 2.40)	3.81 (-1.42, 10.80)
**Czechia**	333.91 (216.02, 473.94)	112.43 (55.78, 176.21)	3.70 (2.40, 5.24)	1.23 (0.60, 1.93)	3.70 (2.40, 5.24)	3.44 (1.90, 6.26)
**Côte d'Ivoire**	22609.60 (11919.46, 31107.12)	5138.54 (1511.41, 8904.62)	253.48 (135.03, 348.29)	57.47 (16.91, 99.23)	253.48 (135.03, 348.29)	57.33 (-8.25, 138.50)
**Democratic People's Republic of Korea**	2756.41 (1946.21, 3591.55)	336.68 (191.55, 533.47)	29.23 (20.26, 38.91)	3.68 (2.11, 5.82)	29.23 (20.26, 38.91)	10.19 (-5.98, 26.40)
**Democratic Republic of the Congo**	29854.26 (11258.92, 42721.19)	4537.62 (152.03, 9078.61)	334.69 (126.55, 478.19)	50.35 (1.54, 101.37)	334.69 (126.55, 478.19)	97.10 (14.57, 190.86)
**Denmark**	83.70 (43.04, 132.81)	21.49 (5.65, 49.89)	0.88 (0.47, 1.36)	0.20 (0.08, 0.34)	0.88 (0.47, 1.36)	3.89 (-1.65, 22.22)
**Djibouti**	20552.43 (16009.00, 25362.56)	3222.00 (2142.54, 4497.31)	228.10 (177.87, 282.58)	33.85 (21.44, 48.22)	228.10 (177.87, 282.58)	235.36 (148.83, 344.54)
**Dominica**	767.50 (483.95, 1055.42)	670.93 (397.45, 1037.37)	8.60 (5.46, 11.83)	7.53 (4.48, 11.64)	8.60 (5.46, 11.83)	2.21 (0.07, 4.99)
**Dominican Republic**	7201.93 (5013.35, 9245.60)	689.43 (379.71, 1063.91)	80.86 (56.59, 103.74)	7.78 (4.29, 12.01)	80.86 (56.59, 103.74)	2.03 (-0.60, 5.54)
**Ecuador**	5389.22 (3820.46, 6515.81)	473.26 (292.00, 716.71)	60.18 (43.46, 72.56)	5.32 (3.32, 8.04)	60.18 (43.46, 72.56)	3.02 (-0.15, 6.85)

**Egypt**	11952.51 (8283.54, 15523.79)	877.72 (556.75, 1195.35)	133.61 (92.95, 173.64)	9.59 (6.06, 13.04)	133.61 (92.95, 173.64)	23.92 (11.36, 41.15)
**El Salvador**	6500.45 (4285.45, 8280.54)	391.95 (232.99, 592.73)	72.78 (48.34, 92.59)	4.40 (2.64, 6.67)	72.78 (48.34, 92.59)	2.47 (-1.02, 6.66)
**Equatorial Guinea**	33047.67 (17774.72, 44879.26)	1890.74 (195.76, 4157.27)	370.86 (199.96, 502.94)	21.11 (2.28, 46.48)	370.86 (199.96, 502.94)	22.94 (-10.77, 79.53)
**Eritrea**	39376.39 (31370.36, 48017.10)	5057.60 (3464.69, 6914.43)	444.13 (354.04, 542.73)	56.34 (38.27, 77.47)	444.13 (354.04, 542.73)	101.04 (60.52, 149.44)
**Estonia**	486.67 (360.55, 615.96)	39.39 (23.97, 61.73)	5.26 (3.96, 6.57)	0.42 (0.28, 0.62)	5.26 (3.96, 6.57)	2.51 (-1.44, 7.63)
**Eswatini**	14229.08 (10135.07, 17704.54)	4376.62 (2879.29, 6187.10)	159.94 (114.67, 198.47)	49.13 (32.43, 69.41)	159.94 (114.67, 198.47)	5.35 (-4.74, 15.95)
**Ethiopia**	40742.98 (32999.59, 48050.70)	4043.80 (2653.83, 5560.72)	459.38 (371.63, 542.75)	45.01 (29.48, 62.15)	459.38 (371.63, 542.75)	77.79 (44.99, 116.20)
**Fiji**	2189.08 (1452.68, 2954.69)	880.48 (474.87, 1456.49)	23.73 (15.57, 32.33)	9.69 (5.23, 16.15)	23.73 (15.57, 32.33)	21.05 (10.04, 36.40)
**Finland**	48.85 (25.15, 80.38)	11.66 (3.58, 21.05)	0.49 (0.28, 0.76)	0.10 (0.05, 0.17)	0.49 (0.28, 0.76)	2.44 (-0.95, 6.08)
**France**	71.19 (39.21, 107.02)	25.12 (8.52, 65.72)	0.75 (0.45, 1.08)	0.20 (0.12, 0.32)	0.75 (0.45, 1.08)	7.25 (-1.76, 40.32)
**Gabon**	9622.52 (4229.73, 14588.96)	1279.53 (351.54, 2501.11)	107.55 (48.15, 163.29)	14.23 (4.03, 27.94)	107.55 (48.15, 163.29)	24.12 (-12.09, 88.21)
**Gambia**	21776.10 (14683.19, 27871.52)	2656.31 (1658.91, 3724.39)	245.71 (166.26, 314.13)	29.73 (18.53, 41.82)	245.71 (166.26, 314.13)	41.33 (23.46, 64.28)
**Georgia**	3584.00 (2463.89, 4824.60)	139.35 (83.31, 227.94)	40.13 (27.69, 53.92)	1.55 (0.94, 2.53)	40.13 (27.69, 53.92)	1.42 (-0.73, 3.47)
**Germany**	68.50 (34.37, 107.98)	23.07 (3.55, 45.02)	0.72 (0.40, 1.09)	0.11 (0.05, 0.18)	0.72 (0.40, 1.09)	13.59 (-0.75, 29.94)
**Ghana**	19776.47 (10064.30, 26791.21)	2406.65 (831.54, 4400.61)	222.08 (114.45, 299.49)	26.84 (9.34, 49.22)	222.08 (114.45, 299.49)	45.32 (8.55, 97.38)
**Greece**	33.59 (16.15, 59.89)	14.28 (4.08, 63.48)	0.34 (0.20, 0.56)	0.11 (0.06, 0.20)	0.34 (0.20, 0.56)	4.06 (-1.04, 46.68)
**Greenland**	679.70 (450.71, 1012.15)	65.70 (37.17, 118.49)	7.61 (5.05, 11.33)	0.72 (0.42, 1.17)	7.61 (5.05, 11.33)	1.92 (-0.15, 14.66)
**Grenada**	1327.79 (938.13, 1768.56)	354.41 (243.71, 492.91)	14.83 (10.52, 19.74)	3.97 (2.72, 5.48)	14.83 (10.52, 19.74)	3.68 (0.32, 8.35)
**Guam**	642.50 (412.66, 948.86)	388.66 (223.03, 636.78)	7.19 (4.68, 10.57)	4.33 (2.52, 7.07)	7.19 (4.68, 10.57)	3.63 (-2.15, 10.58)
**Guatemala**	16064.49 (13089.69, 18566.39)	2979.13 (2063.93, 3978.80)	181.27 (148.43, 209.21)	33.45 (23.21, 44.66)	181.27 (148.43, 209.21)	5.76 (-5.22, 21.97)
**Guinea**	48695.30 (33499.81, 60766.27)	8508.67 (3181.03, 13755.41)	549.28 (378.37, 684.93)	95.30 (35.68, 154.14)	549.28 (378.37, 684.93)	100.52 (10.69, 205.32)
**Guinea-Bissau**	40603.66 (26402.86, 52256.03)	4296.47 (2367.12, 6650.35)	458.30 (299.56, 589.68)	48.31 (26.64, 75.06)	458.30 (299.56, 589.68)	40.26 (15.88, 74.75)
**Guyana**	6032.31 (4079.74, 7767.79)	1017.19 (659.84, 1480.67)	66.29 (44.40, 85.50)	11.19 (7.22, 16.34)	66.29 (44.40, 85.50)	29.14 (15.75, 47.30)
**Haiti**	27484.53 (19456.42, 34245.86)	6615.77 (4025.60, 9224.54)	308.44 (218.56, 384.54)	73.99 (44.98, 103.20)	308.44 (218.56, 384.54)	33.92 (19.93, 53.77)
**Honduras**	6910.37 (4599.49, 8661.78)	590.95 (306.95, 907.17)	77.68 (52.05, 97.28)	6.64 (3.47, 10.18)	77.68 (52.05, 97.28)	2.84 (-2.48, 8.38)
**Hungary**	315.95 (218.48, 412.40)	113.42 (54.28, 175.15)	3.47 (2.42, 4.50)	1.25 (0.61, 1.93)	3.47 (2.42, 4.50)	1.53 (0.08, 3.41)
**Iceland**	49.28 (24.37, 85.51)	18.94 (5.11, 55.74)	0.51 (0.28, 0.87)	0.16 (0.08, 0.28)	0.51 (0.28, 0.87)	4.88 (-1.32, 31.31)
**India**	17597.78 (13722.43, 20717.10)	2255.23 (1613.81, 2931.34)	194.18 (150.99, 229.59)	23.25 (16.26, 30.43)	194.18 (150.99, 229.59)	183.50 (115.84, 266.43)
**Indonesia**	14404.83 (10409.18, 17213.73)	1433.70 (963.92, 1907.35)	159.24 (115.24, 190.98)	14.87 (9.70, 19.93)	159.24 (115.24, 190.98)	112.92 (66.46, 169.42)
**Iran (Islamic Republic of)**	2751.94 (1833.24, 4023.90)	63.29 (40.71, 93.90)	29.69 (19.74, 43.96)	0.56 (0.34, 0.86)	29.69 (19.74, 43.96)	13.88 (7.80, 21.82)

**Iraq**	4335.28 (3125.77, 5732.07)	519.74 (332.48, 741.41)	47.49 (34.31, 62.87)	5.62 (3.59, 8.03)	47.49 (34.31, 62.87)	21.18 (10.27, 36.43)
**Ireland**	84.82 (46.81, 139.39)	20.71 (4.39, 56.09)	0.90 (0.53, 1.46)	0.12 (0.06, 0.21)	0.90 (0.53, 1.46)	9.66 (-1.16, 37.95)
**Israel**	82.64 (42.87, 130.96)	19.76 (4.57, 51.75)	0.89 (0.48, 1.39)	0.15 (0.06, 0.27)	0.89 (0.48, 1.39)	6.39 (-0.73, 28.81)
**Italy**	77.01 (45.09, 119.15)	19.43 (4.87, 42.08)	0.82 (0.50, 1.27)	0.13 (0.06, 0.23)	0.82 (0.50, 1.27)	7.52 (-0.41, 22.34)
**Jamaica**	1765.18 (1236.90, 2262.62)	160.38 (96.68, 237.24)	19.72 (13.88, 25.25)	1.80 (1.09, 2.66)	19.72 (13.88, 25.25)	1.99 (0.02, 4.78)
**Japan**	119.20 (73.84, 159.61)	32.88 (10.87, 55.43)	1.21 (0.86, 1.54)	0.26 (0.16, 0.36)	1.21 (0.86, 1.54)	9.93 (-3.21, 25.36)
**Jordan**	1381.50 (979.21, 1812.18)	180.85 (112.67, 274.11)	15.26 (10.94, 19.94)	2.01 (1.27, 3.04)	15.26 (10.94, 19.94)	2.32 (-0.83, 6.33)
**Kazakhstan**	4612.16 (3210.10, 5850.80)	213.89 (144.02, 303.80)	51.48 (35.96, 65.18)	2.34 (1.56, 3.34)	51.48 (35.96, 65.18)	5.54 (3.02, 8.70)
**Kenya**	16343.46 (10603.49, 20557.81)	3033.18 (1715.29, 4356.74)	183.10 (119.42, 229.57)	33.99 (19.35, 48.88)	183.10 (119.42, 229.57)	30.95 (5.69, 60.65)
**Kiribati**	13200.18 (9484.83, 16862.62)	2981.89 (1909.34, 4165.92)	147.40 (106.14, 188.33)	33.52 (21.47, 46.87)	147.40 (106.14, 188.33)	15.54 (4.94, 28.90)
**Kuwait**	542.16 (345.33, 727.36)	147.47 (83.20, 238.60)	5.86 (3.84, 7.77)	1.63 (0.94, 2.62)	5.86 (3.84, 7.77)	2.05 (-0.96, 5.74)
**Kyrgyzstan**	7355.38 (5108.18, 9156.89)	577.10 (362.70, 846.18)	82.35 (57.38, 102.39)	6.47 (4.08, 9.47)	82.35 (57.38, 102.39)	2.55 (0.27, 5.52)
**Lao People's Democratic Republic**	39401.17 (31691.45, 48680.81)	2960.51 (1795.01, 4101.00)	441.75 (356.29, 546.35)	32.81 (19.99, 45.44)	441.75 (356.29, 546.35)	38.27 (13.68, 64.51)
**Latvia**	389.39 (278.59, 490.97)	44.55 (28.89, 67.55)	4.18 (3.03, 5.23)	0.48 (0.33, 0.71)	4.18 (3.03, 5.23)	1.99 (-0.85, 5.67)
**Lebanon**	2026.73 (1365.48, 2775.55)	259.07 (151.85, 400.89)	22.09 (14.83, 30.45)	2.77 (1.67, 4.29)	22.09 (14.83, 30.45)	12.21 (3.27, 24.63)
**Lesotho**	16282.90 (11928.71, 19845.80)	9028.69 (5953.08, 12332.85)	182.01 (133.80, 222.03)	101.21 (66.93, 138.33)	182.01 (133.80, 222.03)	10.57 (-1.51, 21.92)
**Liberia**	52847.35 (30823.61, 68722.91)	5421.30 (1296.91, 9521.46)	595.40 (349.36, 773.56)	60.61 (14.82, 106.44)	595.40 (349.36, 773.56)	65.98 (-27.27, 178.68)
**Libya**	2055.64 (1346.19, 2886.70)	324.25 (209.59, 463.26)	22.61 (14.73, 31.75)	3.43 (2.19, 4.99)	22.61 (14.73, 31.75)	21.38 (11.12, 36.10)
**Lithuania**	334.11 (233.49, 439.84)	46.30 (28.56, 71.04)	3.59 (2.57, 4.67)	0.50 (0.33, 0.74)	3.59 (2.57, 4.67)	2.24 (-1.39, 6.71)
**Luxembourg**	63.09 (33.15, 101.08)	16.24 (5.04, 28.11)	0.65 (0.37, 1.03)	0.16 (0.07, 0.27)	0.65 (0.37, 1.03)	2.26 (-0.87, 5.84)
**Madagascar**	36027.72 (27624.00, 42034.60)	7526.24 (4533.60, 10502.59)	406.82 (313.48, 474.43)	84.33 (50.82, 118.01)	406.82 (313.48, 474.43)	93.87 (48.16, 147.99)
**Malawi**	45852.54 (24341.49, 60258.73)	4438.77 (2017.90, 6817.09)	516.65 (276.11, 678.39)	49.92 (22.85, 76.65)	516.65 (276.11, 678.39)	37.37 (-9.38, 93.06)
**Malaysia**	1623.95 (1205.10, 2039.30)	258.60 (175.79, 345.69)	16.56 (12.18, 20.86)	2.05 (1.38, 2.75)	16.56 (12.18, 20.86)	76.28 (46.04, 116.12)
**Maldives**	10135.69 (7516.53, 13132.28)	452.43 (303.40, 609.27)	111.69 (83.07, 145.65)	4.51 (3.02, 6.15)	111.69 (83.07, 145.65)	52.91 (26.62, 86.64)
**Mali**	48745.91 (29480.03, 63630.63)	10572.66 (5497.80, 15661.99)	549.59 (332.64, 716.96)	119.08 (61.87, 176.24)	549.59 (332.64, 716.96)	89.26 (12.15, 171.70)
**Malta**	68.98 (37.39, 110.96)	19.85 (6.75, 35.84)	0.71 (0.42, 1.10)	0.18 (0.09, 0.32)	0.71 (0.42, 1.10)	3.38 (-1.29, 8.30)
**Marshall Islands**	3424.23 (2403.95, 4476.35)	1312.40 (706.74, 2055.34)	38.37 (27.07, 50.07)	14.65 (7.90, 22.96)	38.37 (27.07, 50.07)	11.58 (0.32, 24.68)
**Mauritania**	17199.81 (12328.33, 21355.06)	2058.60 (1189.10, 3015.54)	191.92 (137.06, 239.05)	22.33 (12.66, 33.14)	191.92 (137.06, 239.05)	84.63 (52.42, 127.41)
**Mauritius**	1421.32 (1012.50, 1708.55)	408.60 (286.74, 527.05)	13.91 (9.93, 16.47)	3.84 (2.64, 4.99)	13.91 (9.93, 16.47)	66.66 (38.78, 104.37)

**Mexico**	6636.28 (4779.06, 7988.47)	474.20 (295.29, 693.51)	74.24 (53.67, 89.30)	5.31 (3.32, 7.76)	74.24 (53.67, 89.30)	5.90 (2.16, 11.17)
**Micronesia (Federated States of)**	4870.98 (3290.89, 6491.26)	946.84 (597.47, 1419.65)	54.49 (36.90, 72.42)	10.49 (6.58, 15.80)	54.49 (36.90, 72.42)	15.13 (7.12, 25.76)
**Monaco**	52.02 (20.91, 121.85)	29.45 (8.45, 70.84)	0.51 (0.25, 1.00)	0.21 (0.11, 0.39)	0.51 (0.25, 1.00)	10.91 (-0.99, 36.29)
**Mongolia**	14125.13 (10301.33, 17957.17)	610.48 (364.86, 925.70)	158.92 (116.10, 202.11)	6.85 (4.10, 10.37)	158.92 (116.10, 202.11)	0.88 (-0.34, 2.29)
**Montenegro**	267.38 (172.14, 408.57)	28.38 (17.49, 43.87)	2.98 (1.92, 4.54)	0.28 (0.17, 0.45)	2.98 (1.92, 4.54)	3.33 (1.37, 5.99)
**Morocco**	9266.25 (6362.21, 12273.43)	418.83 (219.29, 681.12)	103.52 (71.59, 136.76)	4.65 (2.45, 7.57)	103.52 (71.59, 136.76)	5.26 (1.05, 10.68)
**Mozambique**	42735.25 (24774.31, 56189.22)	4442.59 (1518.24, 7532.26)	481.56 (279.98, 633.25)	49.74 (17.33, 84.31)	481.56 (279.98, 633.25)	46.07 (-16.36, 120.36)
**Myanmar**	27956.30 (20307.21, 35490.72)	2128.19 (1317.99, 2955.50)	313.73 (228.62, 398.64)	23.58 (14.65, 32.69)	313.73 (228.62, 398.64)	29.85 (2.86, 59.71)
**Namibia**	12323.66 (8927.13, 14951.66)	3608.84 (2214.87, 5256.09)	137.69 (99.65, 167.19)	40.29 (24.66, 58.79)	137.69 (99.65, 167.19)	35.11 (19.64, 54.64)
**Nauru**	3393.75 (2295.50, 4728.56)	1745.85 (1056.54, 2777.58)	38.19 (25.97, 53.17)	19.66 (11.94, 31.25)	38.19 (25.97, 53.17)	3.99 (-2.64, 11.80)
**Nepal**	27600.30 (22378.03, 31993.59)	1491.57 (975.51, 2098.62)	309.64 (251.00, 359.11)	16.37 (10.52, 23.19)	309.64 (251.00, 359.11)	38.93 (22.75, 59.67)
**Netherlands**	63.21 (30.09, 129.74)	35.20 (5.73, 78.53)	0.58 (0.32, 0.95)	0.16 (0.08, 0.27)	0.58 (0.32, 0.95)	20.91 (-1.03, 57.62)
**New Zealand**	135.18 (79.34, 212.45)	19.47 (9.25, 44.03)	1.50 (0.90, 2.34)	0.20 (0.11, 0.35)	1.50 (0.90, 2.34)	1.35 (-0.50, 13.75)
**Nicaragua**	9155.43 (5814.98, 11810.90)	545.84 (321.07, 845.23)	102.47 (65.43, 132.00)	6.11 (3.61, 9.44)	102.47 (65.43, 132.00)	2.17 (-1.74, 6.89)
**Niger**	80455.03 (52418.81, 97497.29)	12172.71 (3332.37, 19650.37)	908.23 (592.97, 1102.44)	136.34 (36.98, 220.96)	908.23 (592.97, 1102.44)	160.76 (43.25, 283.28)
**Nigeria**	45841.04 (28137.61, 56750.82)	11646.18 (4644.80, 16983.44)	515.92 (317.64, 638.89)	130.27 (51.93, 189.66)	515.92 (317.64, 638.89)	143.76 (39.21, 266.10)
**Niue**	1924.35 (1225.43, 2827.06)	3378.39 (2148.83, 4968.26)	21.62 (13.84, 31.72)	38.13 (24.34, 55.96)	21.62 (13.84, 31.72)	4.65 (-1.92, 12.44)
**North Macedonia**	2530.98 (1484.47, 3504.51)	114.93 (56.90, 182.73)	28.10 (16.52, 38.85)	1.23 (0.60, 1.96)	28.10 (16.52, 38.85)	5.47 (3.11, 9.05)
**Northern Mariana Islands**	449.37 (270.30, 706.06)	194.56 (125.28, 295.72)	5.02 (3.08, 7.88)	2.16 (1.41, 3.25)	5.02 (3.08, 7.88)	3.71 (-2.21, 11.35)
**Norway**	38.60 (20.05, 63.96)	11.56 (1.86, 30.65)	0.40 (0.23, 0.65)	0.06 (0.03, 0.09)	0.40 (0.23, 0.65)	6.64 (-0.61, 23.01)
**Oman**	1898.58 (1328.83, 2678.71)	248.32 (163.66, 329.07)	19.95 (13.71, 28.53)	2.41 (1.51, 3.25)	19.95 (13.71, 28.53)	34.37 (19.62, 53.87)
**Pakistan**	17014.18 (13225.01, 19814.01)	3244.26 (2201.70, 4531.95)	188.58 (146.49, 219.63)	35.20 (23.55, 49.74)	188.58 (146.49, 219.63)	117.85 (71.41, 176.70)
**Palau**	2295.88 (1295.67, 3609.65)	754.86 (438.80, 1216.43)	25.69 (14.62, 40.21)	8.45 (4.94, 13.60)	25.69 (14.62, 40.21)	3.87 (-1.31, 10.05)
**Palestine**	2507.18 (1686.29, 3459.88)	159.38 (93.93, 250.53)	27.88 (18.80, 38.59)	1.78 (1.06, 2.80)	27.88 (18.80, 38.59)	1.33 (-0.30, 3.55)
**Panama**	2030.42 (1430.50, 2617.21)	652.46 (426.20, 920.65)	22.71 (16.24, 29.16)	7.29 (4.85, 10.18)	22.71 (16.24, 29.16)	5.50 (-4.00, 23.78)
**Papua New Guinea**	17244.31 (12683.12, 22196.08)	6536.99 (4355.69, 8882.16)	191.96 (141.88, 247.03)	71.86 (47.85, 98.18)	191.96 (141.88, 247.03)	141.17 (71.62, 220.00)
**Paraguay**	2202.56 (1413.26, 3020.29)	411.34 (237.43, 617.46)	24.43 (16.07, 33.23)	4.55 (2.66, 6.84)	24.43 (16.07, 33.23)	7.11 (0.40, 15.64)
**Peru**	8802.33 (6497.96, 10942.82)	589.39 (333.10, 935.12)	98.75 (73.57, 122.46)	6.58 (3.75, 10.44)	98.75 (73.57, 122.46)	6.88 (-2.02, 17.24)
**Philippines**	9082.22 (6838.38, 11024.34)	1336.01 (934.93, 1759.42)	101.41 (77.24, 123.52)	14.50 (10.20, 19.22)	101.41 (77.24, 123.52)	53.14 (28.70, 86.66)

**Poland**	293.15 (206.96, 377.31)	55.56 (30.95, 82.15)	3.23 (2.29, 4.13)	0.61 (0.35, 0.91)	3.23 (2.29, 4.13)	0.90 (0.29, 1.90)
**Portugal**	225.76 (135.19, 336.45)	17.80 (7.89, 30.62)	2.45 (1.51, 3.62)	0.18 (0.10, 0.29)	2.45 (1.51, 3.62)	1.93 (-0.86, 5.54)
**Puerto Rico**	278.96 (186.86, 384.08)	86.94 (54.65, 129.06)	3.11 (2.13, 4.25)	0.96 (0.62, 1.41)	3.11 (2.13, 4.25)	1.77 (-0.79, 5.12)
**Qatar**	616.42 (390.59, 878.71)	53.30 (28.97, 84.08)	6.81 (4.38, 9.73)	0.58 (0.33, 0.89)	6.81 (4.38, 9.73)	1.85 (-0.96, 5.44)
**Republic of Korea**	226.57 (139.50, 325.69)	12.61 (5.34, 22.65)	2.52 (1.58, 3.62)	0.14 (0.06, 0.24)	2.52 (1.58, 3.62)	0.62 (-0.32, 1.87)
**Republic of Moldova**	1752.79 (1178.97, 2326.45)	208.29 (122.09, 328.18)	19.39 (13.09, 25.71)	2.31 (1.38, 3.61)	19.39 (13.09, 25.71)	2.75 (-1.02, 7.54)
**Romania**	3652.61 (2554.83, 4578.57)	309.75 (193.90, 438.57)	40.84 (28.61, 51.15)	3.46 (2.17, 4.90)	40.84 (28.61, 51.15)	1.29 (0.00, 2.86)
**Russian Federation**	888.79 (642.17, 1093.53)	97.75 (63.55, 134.47)	9.58 (7.05, 11.68)	1.04 (0.68, 1.42)	130.35 (57.28, 211.90)	5.23 (2.40, 9.43)
**Rwanda**	32033.10 (19683.32, 41703.86)	3186.64 (1636.19, 4975.88)	362.10 (223.16, 471.11)	36.05 (18.59, 56.24)	159.79 (99.08, 236.99)	8.86 (-2.00, 20.98)
**Saint Kitts and Nevis**	2347.79 (1712.95, 2962.17)	675.98 (452.84, 954.47)	26.44 (19.38, 33.32)	7.65 (5.15, 10.77)	133.82 (72.55, 209.23)	2.44 (-0.28, 5.95)
**Saint Lucia**	1214.11 (838.38, 1598.32)	262.96 (156.02, 414.36)	13.51 (9.38, 17.81)	2.93 (1.74, 4.62)	5.23 (-3.58, 15.68)	5.15 (0.86, 11.57)
**Saint Vincent and the Grenadines**	2238.27 (1555.85, 2904.94)	480.17 (329.75, 684.82)	25.15 (17.58, 32.63)	5.47 (3.76, 7.76)	3.75 (-1.14, 9.89)	2.69 (0.39, 5.59)
**Samoa**	2582.50 (1738.52, 3603.46)	641.98 (372.16, 1010.52)	28.57 (19.06, 40.04)	6.99 (3.98, 11.11)	206.79 (30.88, 388.82)	21.01 (11.76, 33.10)
**San Marino**	53.91 (27.36, 98.99)	10.00 (1.09, 39.29)	0.57 (0.31, 1.04)	0.05 (0.02, 0.09)	60.71 (37.40, 91.81)	5.54 (-1.08, 31.71)
**Sao Tome and Principe**	23089.03 (18283.74, 27349.98)	950.86 (568.97, 1478.97)	259.95 (206.49, 307.65)	10.59 (6.31, 16.52)	10.35 (-6.42, 29.11)	14.97 (8.19, 23.87)
**Saudi Arabia**	3021.12 (2039.93, 4144.37)	109.52 (68.00, 167.13)	31.90 (21.13, 44.32)	0.82 (0.43, 1.34)	16.35 (-3.38, 39.37)	37.42 (21.57, 60.52)
**Senegal**	25421.63 (16166.31, 31850.80)	2161.41 (1039.38, 3347.79)	286.89 (182.98, 359.55)	24.14 (11.63, 37.44)	0.81 (-0.49, 2.60)	28.35 (10.00, 51.35)
**Serbia**	667.75 (450.02, 943.84)	49.93 (30.01, 71.88)	7.44 (5.04, 10.53)	0.51 (0.30, 0.75)	8.62 (-2.64, 23.62)	4.09 (2.04, 6.96)
**Seychelles**	1066.13 (769.54, 1375.19)	448.10 (277.15, 662.80)	11.95 (8.68, 15.38)	4.98 (3.10, 7.33)	72.24 (37.48, 114.21)	4.72 (-0.79, 12.01)
**Sierra Leone**	51331.90 (31914.58, 66107.49)	11446.52 (4914.71, 18576.03)	577.98 (360.11, 743.05)	128.75 (55.60, 209.28)	4.48 (0.00, 10.17)	86.25 (-2.77, 209.16)
**Singapore**	409.32 (259.60, 544.51)	24.56 (12.87, 40.64)	4.40 (2.81, 5.83)	0.27 (0.15, 0.44)	57.68 (33.49, 92.72)	0.57 (-0.09, 1.93)
**Slovakia**	748.23 (531.51, 962.35)	186.25 (120.00, 265.60)	8.35 (5.93, 10.74)	2.07 (1.33, 2.95)	290.30 (160.17, 439.33)	1.80 (0.72, 3.09)
**Slovenia**	323.06 (236.04, 435.75)	49.45 (30.46, 72.59)	3.59 (2.64, 4.84)	0.54 (0.35, 0.79)	20.83 (10.09, 36.16)	1.57 (-0.33, 3.98)
**Solomon Islands**	7874.02 (5548.08, 10166.44)	1987.69 (1341.42, 2699.13)	87.47 (62.02, 113.03)	21.61 (14.52, 29.12)	7.52 (-2.25, 19.39)	67.80 (33.08, 115.07)
**Somalia**	44056.73 (33228.06, 53569.27)	13909.88 (7752.60, 19998.43)	496.00 (373.58, 603.19)	155.77 (85.89, 225.52)	4.08 (-0.25, 9.94)	153.01 (95.90, 222.37)
**South Africa**	13496.17 (9899.19, 16024.64)	3311.32 (2235.88, 4357.03)	150.98 (111.38, 179.18)	37.05 (25.11, 48.71)	30.12 (9.81, 60.58)	14.63 (3.37, 28.46)
**South Sudan**	50639.62 (35443.62, 62934.97)	19242.92 (10323.49, 27630.93)	567.54 (397.69, 705.84)	213.98 (114.31, 309.68)	182.97 (21.21, 364.03)	327.29 (194.30, 488.60)
**Spain**	54.04 (31.42, 83.83)	10.74 (2.60, 19.86)	0.57 (0.38, 0.86)	0.10 (0.04, 0.16)	2.97 (-1.21, 7.90)	2.20 (-1.20, 6.64)
**Sri Lanka**	1716.68 (1160.24, 2099.19)	248.58 (177.86, 338.27)	17.08 (11.53, 21.10)	1.49 (0.93, 2.22)	160.77 (-89.15, 413.26)	115.92 (73.73, 173.88)

**Sudan**	25012.30 (17849.40, 32563.12)	1631.44 (830.96, 2692.93)	278.67 (199.04, 363.25)	16.95 (7.91, 28.74)	37.26 (-25.49, 95.92)	131.67 (77.51, 200.29)
**Suriname**	4083.97 (2563.89, 5404.22)	832.77 (452.88, 1257.85)	45.20 (28.34, 60.01)	9.15 (4.92, 13.90)	14.02 (7.34, 22.88)	18.71 (10.14, 29.27)
**Sweden**	41.35 (21.43, 69.47)	13.16 (2.85, 40.85)	0.43 (0.24, 0.72)	0.08 (0.04, 0.14)	96.29 (37.40, 168.74)	5.80 (-0.59, 28.94)
**Switzerland**	67.39 (32.35, 113.12)	17.66 (2.44, 49.73)	0.70 (0.38, 1.13)	0.11 (0.05, 0.19)	26.08 (-3.15, 59.00)	8.17 (-1.90, 35.06)
**Syrian Arab Republic**	3885.44 (2879.44, 5239.55)	421.98 (308.15, 570.96)	41.71 (30.72, 56.94)	3.51 (2.36, 5.06)	9.87 (4.92, 17.97)	112.90 (69.42, 165.43)
**Taiwan (Province of China)**	420.48 (263.13, 576.92)	61.23 (29.38, 98.78)	4.62 (2.98, 6.28)	0.62 (0.37, 0.92)	10.26 (5.79, 15.45)	6.55 (-3.31, 18.78)
**Tajikistan**	17338.79 (12113.42, 20725.66)	3994.67 (2563.36, 5503.25)	193.95 (135.59, 232.10)	44.36 (28.26, 61.26)	391.18 (28.21, 731.57)	46.39 (27.99, 70.77)
**Thailand**	2485.15 (1637.56, 3234.46)	392.85 (256.42, 516.25)	27.20 (18.08, 35.55)	4.09 (2.64, 5.38)	208.70 (-29.10, 422.33)	29.75 (16.35, 48.47)
**Timor-Leste**	34545.92 (25075.39, 43550.48)	3728.86 (2670.76, 4834.01)	385.57 (280.38, 486.70)	39.51 (28.07, 51.90)	48.74 (-6.07, 107.87)	221.71 (142.44, 321.28)
**Togo**	25103.04 (14152.11, 33531.28)	4760.17 (1540.13, 7585.64)	282.55 (160.90, 377.21)	53.34 (17.49, 84.69)	270.89 (136.76, 420.20)	52.79 (-14.41, 131.08)
**Tokelau**	3326.26 (2196.38, 4605.25)	4464.21 (2840.48, 6508.63)	37.41 (24.78, 51.74)	50.37 (32.14, 73.35)	115.12 (-28.94, 272.03)	4.30 (-0.84, 10.22)
**Tonga**	1957.71 (1294.31, 2820.61)	639.74 (390.02, 1020.23)	21.99 (14.61, 31.59)	7.01 (4.22, 11.33)	2.62 (-1.29, 7.12)	18.33 (10.33, 30.55)
**Trinidad and Tobago**	959.69 (663.32, 1291.45)	211.47 (131.34, 309.74)	10.61 (7.30, 14.27)	2.11 (1.25, 3.19)	197.17 (-30.02, 421.53)	24.18 (13.78, 38.36)
**Tunisia**	2954.10 (1903.52, 4440.60)	157.47 (89.57, 245.31)	32.66 (21.05, 49.11)	1.72 (0.97, 2.66)	300.56 (104.38, 504.24)	4.78 (0.62, 10.40)
**Turkey**	5416.95 (3573.50, 7959.09)	136.52 (81.69, 226.06)	60.55 (40.15, 88.81)	1.52 (0.93, 2.44)	3.44 (-2.33, 10.63)	2.41 (-0.89, 14.08)
**Turkmenistan**	13648.69 (9440.93, 16875.70)	1936.85 (1307.07, 2757.35)	152.37 (105.63, 188.49)	21.60 (14.56, 30.71)	33.67 (7.89, 63.14)	13.55 (7.88, 21.99)
**Tuvalu**	9246.36 (6842.19, 12289.30)	983.30 (647.56, 1443.70)	103.67 (76.79, 137.75)	11.04 (7.29, 16.14)	13.72 (-10.39, 38.98)	3.05 (-1.53, 13.01)
**Uganda**	29806.75 (13499.83, 42937.44)	4782.39 (488.75, 9835.64)	335.46 (153.44, 483.02)	53.73 (5.52, 110.56)	216.53 (90.73, 386.51)	40.26 (-3.59, 95.51)
**Ukraine**	608.32 (463.88, 751.63)	175.60 (133.05, 224.05)	5.67 (4.27, 6.99)	1.11 (0.84, 1.42)	111.19 (-14.77, 257.09)	76.98 (46.50, 116.29)
**United Arab Emirates**	1077.91 (764.66, 1423.65)	161.09 (94.18, 240.07)	9.61 (6.62, 12.74)	1.06 (0.66, 1.50)	4.89 (-3.58, 14.99)	66.90 (32.70, 116.13)
**United Kingdom**	59.95 (32.48, 105.97)	13.42 (4.70, 29.75)	0.63 (0.36, 1.09)	0.10 (0.06, 0.20)	6.78 (-3.46, 25.96)	4.15 (-0.34, 12.85)
**United Republic of Tanzania**	32212.69 (18406.80, 41485.74)	4339.11 (2291.60, 6497.95)	361.96 (207.38, 465.32)	48.88 (25.99, 73.08)	3.39 (1.25, 6.23)	20.92 (-3.89, 47.51)
**United States of America**	86.23 (48.38, 140.31)	39.04 (22.73, 63.08)	0.89 (0.55, 1.32)	0.27 (0.17, 0.41)	3.52 (-0.71, 8.98)	15.60 (7.62, 27.20)
**United States Virgin Islands**	459.13 (294.79, 660.73)	93.51 (53.18, 144.60)	5.15 (3.34, 7.40)	1.05 (0.61, 1.61)	7.21 (0.83, 15.89)	1.57 (-0.33, 4.16)
**Uruguay**	967.57 (689.30, 1206.99)	126.47 (80.61, 183.98)	10.74 (7.76, 13.35)	1.41 (0.91, 2.04)	3.70 (1.42, 7.71)	1.23 (-0.80, 3.74)
**Uzbekistan**	8922.97 (6602.62, 10636.91)	1115.64 (762.85, 1601.07)	99.20 (73.35, 118.35)	12.48 (8.53, 17.91)	179.62 (-19.34, 394.53)	5.05 (2.70, 8.79)
**Vanuatu**	4777.10 (3339.31, 6318.80)	1579.79 (1003.45, 2288.58)	52.94 (36.82, 70.02)	17.62 (11.14, 25.60)	157.23 (93.88, 239.64)	15.94 (2.54, 31.14)
**Venezuela (Bolivarian Republic of)**	3574.57 (2302.07, 4424.83)	766.09 (448.34, 1146.11)	39.93 (25.88, 49.34)	8.59 (5.06, 12.87)	238.58 (5.72, 470.46)	4.59 (-2.14, 12.78)
**Viet Nam**	4681.87 (3605.16, 5760.25)	343.66 (203.56, 485.32)	51.18 (39.41, 62.91)	3.51 (2.12, 5.00)	4.97 (-0.25, 12.28)	31.42 (12.43, 56.54)

**Yemen**	23617.78 (17149.36, 29187.48)	1965.41 (1369.36, 2663.12)	262.57 (190.73, 325.01)	20.73 (14.00, 28.40)	372.41 (223.10, 563.53)	125.20 (74.43, 188.01)
**Zambia**	34712.05 (23333.52, 43180.87)	3955.35 (2057.38, 6297.37)	390.78 (263.66, 485.87)	44.47 (23.33, 70.91)	5.33 (0.63, 10.83)	33.12 (-3.08, 78.60)
**Zimbabwe**	8809.40 (6033.08, 11026.94)	6014.23 (4056.78, 8085.20)	98.81 (67.97, 123.53)	44.48 (30.87, 59.86)	19.90 (-9.18, 51.83)	40.53 (21.83, 64.21)
